# Imbalance between muscle strength development and weight gain in children and young adults in China: serial cross-sectional evidence from 1.33 million students from five successive national surveys between 2000 and 2019

**DOI:** 10.1016/j.lanwpc.2025.101640

**Published:** 2025-07-25

**Authors:** Xijie Wang, Huan Wang, Xin Yuan, Shan Cai, Yangmu Huang, Yi Song, Zhiyong Zou, Randall S. Stafford

**Affiliations:** a Vanke School of Public Health, Tsinghua University, Beijing, 100084, China; b Institute for Healthy China, Tsinghua University, Beijing, 100084, China; c Institute of Child and Adolescent Health, Peking University School of Public Health/National Commission of Health Key Laboratory of Reproductive Health, Peking University, Beijing, 100191, China; d Institute for Global Health and Development, Peking University School of Public Health, Peking University, Beijing, 100191, China; e Stanford Prevention Research Center, Palo Alto, CA, 94304, United States

**Keywords:** Muscle strength, Physical fitness, Handgrip strength, Standing broad jump, Student health, Epidemiology, Trend analysis

## Abstract

**Background:**

Over the past decades, Chinese school-aged children and adolescents have experienced a rapid increase in overweight and obesity, but corresponding changes in muscle strength remain unclear. We aimed to elucidate trends in muscle strength development at both national and subnational level, and to investigate whether it has increased proportionally with weight gain.

**Methods:**

Muscle strength (handgrip strength [kg] for upper body and standing broad jump [cm] for lower body) and weight measurements for 1.33 million children and adolescents aged 7 to 22 were obtained from five waves of the Chinese National Surveys on Students Constitution and Health (2000, 2005, 2010, 2014, and 2019). Trends in muscle strength indicators were investigated using t-test, with post-hoc pairwise comparisons performed using Bonferroni correction. Associations between muscle indicators with weight change were assessed with the Pearson correlation analysis.

**Findings:**

Despite some increase during the first waves of survey, both handgrip strength and standing broad jump declined significantly between 2010 and 2019. The median handgrip strength decreased from 43.9 kg (95% CI 43.7 to 44.1) in 2010 to 42.5 kg (42.3 to 42.8) in 2019 among young males, and from 26.6 kg (26.5 to 26.7) in 2010 and to 26.0 kg (25.8 to 26.1) in 2019 among young females. From 2010 to 2019, median standing broad jump decreased from 234.3 cm (233.8–234.8) to 219.4 cm (218.8–220.1) among males, and from 172.8 cm (172.3–173.3) to 162.5 cm (162.0–163.1) among females. Over the last decade, both measures of muscle strength showed significant declines within each age- and sex-specific quantiles, with the most pronounced decreases observed at the lower quantiles and older ages. The most notable declines were observed in North China, Northeast China, and Middle-West China. As age and weight increased, the initially positive association between body weight and handgrip strength shifted to a progressive weakening, while the association between body weight and standing broad jump changed from non-significant to negative, at −0.51 (95% CI: −0.62 to −0.38) for males and −0.39 (95% CI: −0.52 to −0.24) for females.

**Interpretation:**

This nationally representative study highlights a significant decline in muscle strength among Chinese students over the last decade. The imbalance between increasing body weight and declining muscle strength underscores the urgent need for targeted public health interventions to decrease obesity in Chinese students, to maximize their life-long health expectancy.

**Funding:**

This study was supported by the 10.13039/501100001809National Natural Science Foundation of China (82073573 to ZZ; 82204067 to XW; 82273654 to YS).

**Research in context**

**Evidence before this study**

We searched PubMed on January 10th 2025, using the broad terms (“muscle strength” OR “handgrip strength” OR “standing broad jump”) AND (“weight gain” OR “obesity” OR “overweight”) AND (“children” OR “adolescents” OR “students” OR “youth”) AND (“China” OR “Chinese”), with no language restrictions. Two systematic analysis of multiple countries reported that adolescents’ handgrip strength performance generally improved during the early 21st century, while standing broad jump performance declined at accelerating rates. Available Asian data in these studies remain limited, with the most recent evidence covering only up to 2014 (1992–2014). Pooled estimates suggesting that declines in muscle strength among Asian children and adolescents were more pronounced than global averages. In China, while studies confirmed a rapid and ongoing rise in overweight and obesity among school-aged youth—unlike plateauing trends observed in Western countries—evidence on concurrent changes in muscle strength remained scarce. Critically, no studies systematically quantified national trends and subnational disparities in muscle strength or evaluated the decoupling of weight gain from muscle strength in China, leaving a gap in understanding how these dual challenges interact to impact health outcomes.

**Added value of this study**

This serial cross-sectional analysis of 1.33 million children and adolescents aged 7–22 years, spanning five national surveys from 2000 to 2019, provides the first comprehensive assessment of muscle strength trends (via handgrip strength and standing broad jump) alongside weight changes in China. We demonstrate that while weight increased steadily across all demographics, upper- and lower-body muscle strength significantly declined after 2010, with the steepest reductions observed among 18–22-year-olds—an age at which peak muscle strength is expected. Notably, declines were most pronounced in North, Northeast, and Central China, regions with high obesity prevalence, and at lower quantiles of muscle strength, exacerbating health inequities. Critically, we reveal a growing mismatch between rising weight and declining muscle strength, with weight gains only weakly associated with handgrip strength improvements and negatively linked to lower-body strength.

**Implications of all the available evidence**

The findings underscore an urgent need to integrate muscle strength training into national obesity prevention strategies. The disproportionate decline in muscle strength relative to weight gain, particularly in older adolescents, signals a looming public health crisis. Without interventions, this mismatch may amplify risks of musculoskeletal disorders, metabolic syndromes, and reduced functional capacity in middle and older adulthood. Regional disparities highlight the necessity for tailored policies, such as school-based strength training programs in North and Northeast China, where declines are most severe. Existing evidence demonstrate that prevailing obesity interventions—overly reliant on dietary modification and aerobic activity—lack effectiveness while neglecting the essential role of muscular fitness; enhancing muscle strength must become a parallel priority to safeguard long-term population health. This study provides a baseline for policymakers to refocus physical health metrics, monitor progress, and allocate resources to regions and demographics at greatest risk.

## Introduction

Muscle strength is a key component of physical fitness that plays a crucial role in the growth and development of children and adolescents, as well as overall health throughout the lifespan.^1^, ^2^, ^3^ In children and adolescents, muscular strength is independently associated with cardiometabolic health, regardless of weight status and aerobic capacity.^4^, ^5^, ^6^ Additionally, a lower muscular fitness in childhood is linked to an increased risk of non-communicable diseases in adulthood.^4^^,^^7^ Thus, building and maintaining muscular strength early in life is essential for fostering healthy aging, which includes reduced all-cause mortality, improved physical functioning, enhanced cardiometabolic health, and better psychosocial well-being.^8^^,^^9^

Handgrip strength (HGS) and standing broad jump (SBJ), although they may not reflect the whole body muscular capacity or muscular endurance, are widely recognized as reliable indicators of upper and lower body muscular strength in children and adolescents, respectively.^9^, ^10^, ^11^ Handgrip strength measures maximal isometric grip force, reflecting the ability of a muscle or group of muscles to generate maximum force in a single contraction and is considered an excellent marker of overall strength and health across different populations.^12^^,^^13^ Standing broad jump, on the other hand, assesses lower limb muscular fitness and explosive muscular strength in children, adolescents, and adults and is regarded as the most valid and reliable field-based test for muscular fitness,^14^ and also corresponds to lower limb anaerobic power.

With the rapid global rise in childhood overweight and obesity,^15^, ^16^, ^17^ the muscular strength profile of children and adolescents has become increasingly concerning. A recent study involving over 10 million children and adolescents revealed that while standing broad jump performance demonstrated negligible improvement from the 1960s to the 1990s, it has declined significantly since the late 1990s.^18^ Approximately 62% of the countries included in the study experienced this downturn, with the decline particularly notable in Asian countries such as South Korea and China since 2000. In contrast, global trends in handgrip strength generally show improvement, with a 19.4% increase observed between 1967 and 2017, particularly in recent years. However, countries such as China, Bulgaria, South Korea, and New Zealand have reported decreases in handgrip strength for the past few years.^19^^,^^20^ Collectively, these trends highlight a worrisome decline in physical fitness, especially in Asia, where contemporary evidence on muscular strength is limited.

Unlike many Western countries, where childhood obesity rates have plateaued,^21^ China has experienced a continued, dramatic rise in the prevalence of overweight and obesity during the 21st century.^22^ The simultaneous increase in weight gain and decline in muscular strength pose a dual threat to the long-term health of Chinese children and adolescents.^23^ In 1990, Blimkie et al. firstly reported lower quadriceps femoris muscle activation in obese compared to non-obese male adolescent, indicating an association between weight gain and impaired muscle health.^24^ The study also implied that relative to non-obese adolescents, obese adolescents had poorer neural activation capacity, which may likely lead to a reduction in the degree and/or pattern of muscle fiber recruitment. However, subsequent studies in 2000s remained controversial and inconclusive about whether obesity was directly associated to muscle strength in large-scale general population.^25^^,^^26^ Notably, recent decades have seen a lack of longitudinal research examining trends in muscular strength and its association with weight changes in this population,^27^ with only one study of four-year follow-up study reports a persistent association between obesity (but not overweight) and reduced muscle strength.^28^

As children and adolescents aged 7 to 22 generally undergo significant weight gain and muscle strength decline, this study utilizes 20 years of data, collected across five waves (2000, 2005, 2010, 2014, and 2019) of the Chinese National Surveys on Students Constitution and Health (CNSSCH), to examine trends and regional disparities in muscle strength development at the national and subnational levels, and to explore the discrepancy between weight gain and muscular strength changes.

## Methods

### Study design and participants

The CNSSCH, so far, is the largest representative national survey on health status of students in China. The survey is conducted every five years, with a multistage stratified cluster sampling design and has remained consistent approaches to sampling and assessment across all the survey years. For each survey wave, each province was treated as an independent subpopulation, with provincial school health institutes collecting a representative sample from the entire province. Schools were selected evenly based on the affluence levels (upper, moderate, and low) of the prefecture-level cities in which they were located. This ensured a highly consistent sampling approach within each province, maintaining uniformity across all regions. Participants were identified using stratified cluster sampling, with clusters randomly selected from grades 1 to 12 in the chosen primary and secondary schools and universities. The sampling and investigation protocols were consistent across all waves, as previously reported.^10^^,^^17^ Eligible participants and their families needed to have lived locally for at least 1 year, and all measurements followed the same procedures with the same type of apparatus.^29^

For the present retrospective analysis, data were extracted from five successive cycles (2000, 2005, 2010, 2014, and 2019) of the CNSSCH surveys. The main analysis included participants of Han ethnicity from 30 provinces, autonomous regions, and municipalities in mainland China (referred to as provinces hereafter). Data from Hong Kong, Macau, and Taiwan were not included, and the sample from Tibet was excluded due to its predominantly minority ethnic composition. Participants with incomplete data regarding sex, age, setting, region, height, or body weight were also excluded (<1%). The final sample comprised 1,339,060 participants. Participants aged <18 are referred to as “children”, and those aged 18–22 as young adults.

### Procedures

All of the measurements in the CNSSCH followed standardized procedure and were completed by trained professional staff. All the instruments were calibrated before use each day, and their models were similar at each survey site during each survey cycle.^10^^,^^22^^,^^29^

Upper-body muscular strength was assessed with handgrip test, and was measured by an electronic grip strength meter (WCS-100, Beijing Xindong Huateng sports Facilities Co., Ltd, China). The participant used the dominant hand to hold the grip strength meter inside and outside the grip handle, with the other hand turn the grip distance adjustment wheel, adjusted to the appropriate force grip distance. During the test, the participants were asked to keep their body upright, feet naturally separated, shoulder width apart, and both arms diagonally down, palm inward, with the maximum force grip inside and outside the grip. Handgrip strength was measured twice for each person, the maximum value was recorded in kilograms to the nearest 0.1 kg.

Lower-body muscular strength was assessed by standing broad jump, and was measured on a sand pit level with the ground or on a flat surface with soft soil, under instructions of physical education teachers. During the test, the participants should have their feet naturally separated, standing behind the starting line, with both feet jump in place at the same time. A measuring tape, calibrated with a 100 cm steel ruler before each use, was used to measure the vertical distance between the back edge of the jump line and the back edge of the nearest landing spot. Standing broad jump was conducted three times and the best score was recorded in centimeters to the nearest centimeter.

Weight was measured to the nearest 0.1 kg with a standardized scale (electronic weighing scale or lever scale) recording the mean of three measurements. Before each use, the scale was calibrated for sensitivity with 100 g standard weights and accuracy with 10/20/30 kg standard weights. Participants were required to wear light clothing and stand erect, barefoot, and at ease while being measured. Height was measured to the nearest 0.1 cm with portable stadiometers. Similar instruments were used at each survey site, and were calibrated with a 10 cm steel ruler before each use.

The 30 provinces included in our present analysis were grouped into seven geographic regions, including East (Shanghai, Jiangsu, Zhejiang, Anhui, Fujian, Jiangxi, Shandong), Centre (Henan, Hubei, Hunan), South (Guangdong, Guangxi, Hainan), Southwest (Chongqing, Sichuan, Guizhou, Yunnan), North (Beijing, Tianjin, Hebei, Shanxi, Inner Mongolia), Northeast (Liaoning, Jilin, Heilongjiang), and Northwest (Shaanxi, Gansu, Qinghai, Ningxia, Xinjiang) China according to codes for the administrative divisions of the People’s Republic of China (GB/T 2260).^30^

### Statistical analysis

Handgrip strength, standing broad jump, and weight were presented as means with 95% confidence intervals (*CI*), by survey year, sex, age, and province. To present the trends in handgrip strength, standing broad jump, and weight, absolute changes in mean values of these indices were calculated for four year-intervals: 2000–2005, 2005–2010, 2010–2014, 2014–2019. [We analyzed national-level data to assess absolute changes in handgrip strength, standing broad jump, and weight across two time periods: 2000–2010 and 2010–2019. For each sex and age group, the mean differences for each indicator between the two timepoints were compared using t test]. Post-hoc pairwise comparisons were performed with Bonferroni correction. To explore inequalities between muscle strength and weight, the associations between muscle strength and weight from 2000 to 2019 were examined using Pearson correlation analysis for all participants. Age- and sex-specific analyzes were conducted to avoid potential influence and confounding from these factors. Since muscle strength indicators may index with other body measurements, we also introduced the metrics of handgrip strengths (in kg) divided by height (in meters squared [m^2^]), based on recommendations from previous large-scale population-based study,^13^ as a complement to absolute values of muscle strength.

All data analyses and visualizations were carried out using R version 4.4.2 (Boston MA). All statistical tests were two-sided, and a significance level of *P* < 0.05 was used to assess statistical significance.

### Role of the funding source

The funders of the study had no role in study design, data collection, data analysis, data interpretation, or writing of the report.

### Ethics approval

Both children and parents provided written informed consent before participation. The study was approved by the Medical Research Ethics Committee of the Peking University Health Science Center (IRB00001052-19095).

## Results

### Basic demographics

Across five surveys conducted in 2000, 2005, 2010, 2014, and 2019, a total of 1.33 million students aged 7–22 were included in the final analysis, with 670,794 males and 668,266 females. In 2000, the proportions of students aged 7, 12, 18, and 22 years were 6.79%, 6.78%, 6.86%, and 4.10%, respectively, and these in 2019 were 7.04%, 6.99%, 6.43%, 3.90%, respectively (Supplementary Table S1).

### National trends in handgrip strength, standing broad jump, and body weight

**Fig. 1:**
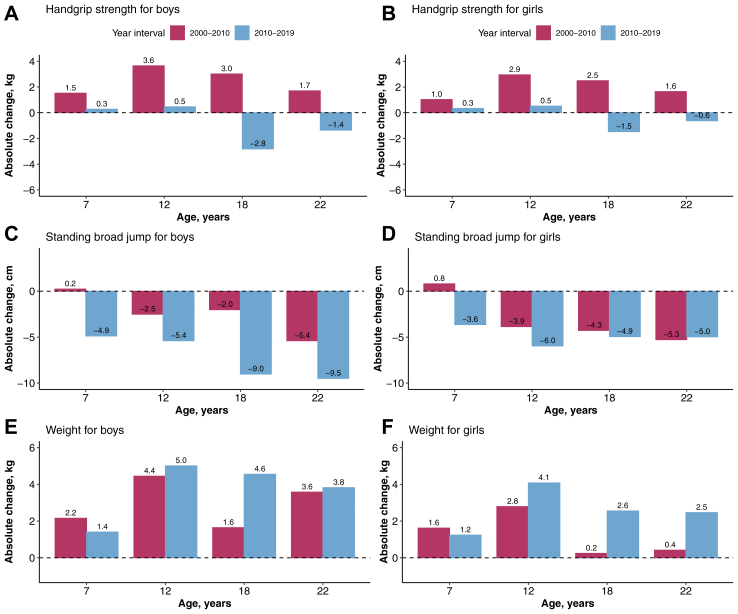
Absolute changes in handgrip strength (A, B), standing broad jump (C, D), and weight (E, F) by age and sex in mainland China, in 2000–2010 and 2010–2019.

In 2019, the mean handgrip strength for males aged 7–22 years was 28.5 kg (95% CI: 28.4 to 28.6), an increase from 27.4 kg (27.3–27.4) in 2000. In 2019, female participants had a mean handgrip strength of 20.2 kg (20.2–20.3), whereas in 2000, they had a mean handgrip strength of 18.7 kg (18.7–18.8). With the exception of Southwest China, where handgrip strength continued to decrease, the other regions followed consistent trend (Supplementary Table S1). The changes of indexed handgrip strength followed similar trends as the absolute value of handgrip strength (Supplementary Table S2). These overall increases were mainly driven by substantial gains in handgrip strength from 2000 to 2010, particularly among children and adolescents aged 12 and 18 years. However, there were significant declines in handgrip strength for adolescents and young youths (individuals aged 18 and 22 years) between 2010 and 2019. For participants aged 22, the median handgrip strength decreased from 43.9 kg (43.7–44.1) in 2010 to 42.5 kg (42.3–42.8) in 2019 among young adult male, and from 26.6 kg (26.5–26.7) in 2010 and to 26.0 kg (25.8–26.1) in 2019 among young adult female (Fig. 1A and B; Supplementary Fig. S1A and B). Absolute changes in handgrip strength over time were displayed in Supplementary Fig. S2A and B.

In 2019, the mean standing broad jump for males aged 7–22 years was 183.1 cm (95% CI 182.9 to 183.3), a decrease from 193.3 cm (193.1–193.5) in 2000. For female counterparts, the mean standing broad jump decreased from 157.8 cm (157.7–158.0) in 2000 to 149.2 cm (149.1–149.3) in 2019 (Supplementary Table S1). From 2000 to 2019, the standing broad jump generally continued to decline among all age groups for both male and female, with particularly large declines in the most recent decade, despite small increases observed from 2000 to 2010 for those aged 7 years. For young adults aged 22, the standing broad jump continued to decrease from 234.3 cm (233.8–234.8) to 219.4 cm (218.8–220.1) in male, and from 172.8 cm (172.3–173.3) to 162.5 cm (162.0–163.1) in female between 2000 and 2019 (Fig. 1C and D; Supplementary Fig. S1C and D). Absolute changes in standing broad jump over time were displayed in Supplementary Fig. S2C and D.

In 2019, the mean body weight for male students aged 7–22 years was 52.4 kg (95% CI 52.3 to 52.5), an increase from 45.7 kg (45.6–45.8) in 2000, and 48.7 kg (48.6–48.8) in 2010. Compared with 41.4 kg (41.4–41.5) in 2000 and 43.0 kg (42.99–43.1) in 2010, females aged 7–22 years had a mean body weight of 45.8 kg (45.8–45.9) in 2019 (Supplementary Table S1). From 2000 to 2019, the mean body weight showed upward trends across all age groups, with a 10%–15% increase in the mean body weight in 2019 compared with 2000 (Fig. 1E and F; Supplementary Fig. S1E and F). Absolute changes in weight over time were displayed in Supplementary Fig. S2E and F.

The above changes were most significant in the 2010–2019 interval, with the greatest change especially in the 18 and 22 year old age groups (*P*_difference_ < 0.001 compared with changes in 7-year-old groups; Supplementary Table S3).

**Fig. 2:**
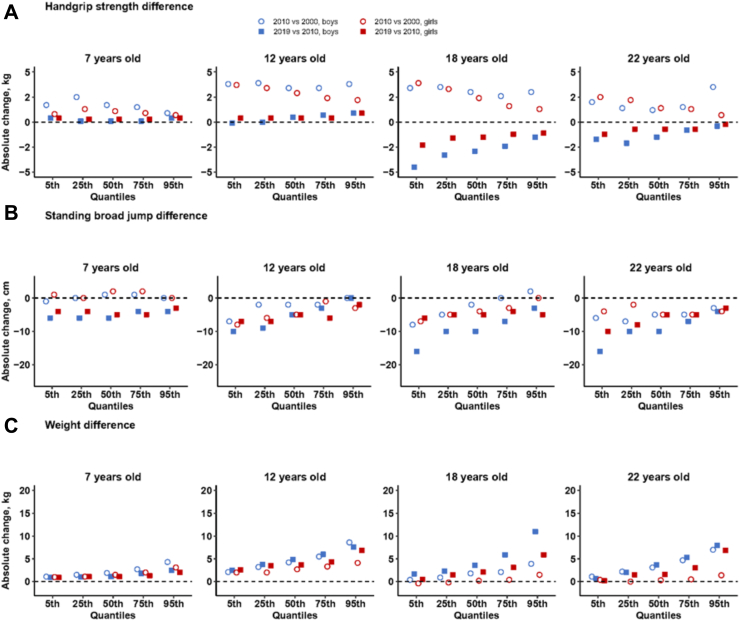
Absolute changes in different quantiles of handgrip strength (A), standing broad jump (B), and weight (C) by age and sex in mainland China, in 2000–2010 and 2010–2019.

Between 2000 and 2010, for male students aged 7 and 12 years, the handgrip strength at all quantiles (5th, 25th, 50th, 75th, and 95th) increased, with the most prominent increase seen among boys aged 12. In contrast, those aged 18 and 22 years showed increased handgrip strength only at 75th and 95th quantiles. For females, the age-specific handgrip strength at all quantiles increased for all age groups, especially among girls aged 12 years. However, from 2010 to 2019, the handgrip strength decreased at all quantiles for both males and females aged 18 and 22 years (Fig. 2A). The age-specific standing broad jump decreased consistently at all quantiles between 2000 and 2010, and between 2010 and 2019, for all age groups and both sexes. Greater decreases were found in those aged 18 and 22 years than younger children aged 7 and 12 years (Fig. 2B). Conversely, the age-specific body weight increased at all quantiles in 2000–2010 and 2010–2019 for all age groups and both sexes. The absolute increases in body weight were greater at higher quantiles, with the largest increase at the 95th quantile (Fig. 2C).

### Regional disparities in handgrip strength, standing broad jump, and weight

**Fig. 3:**
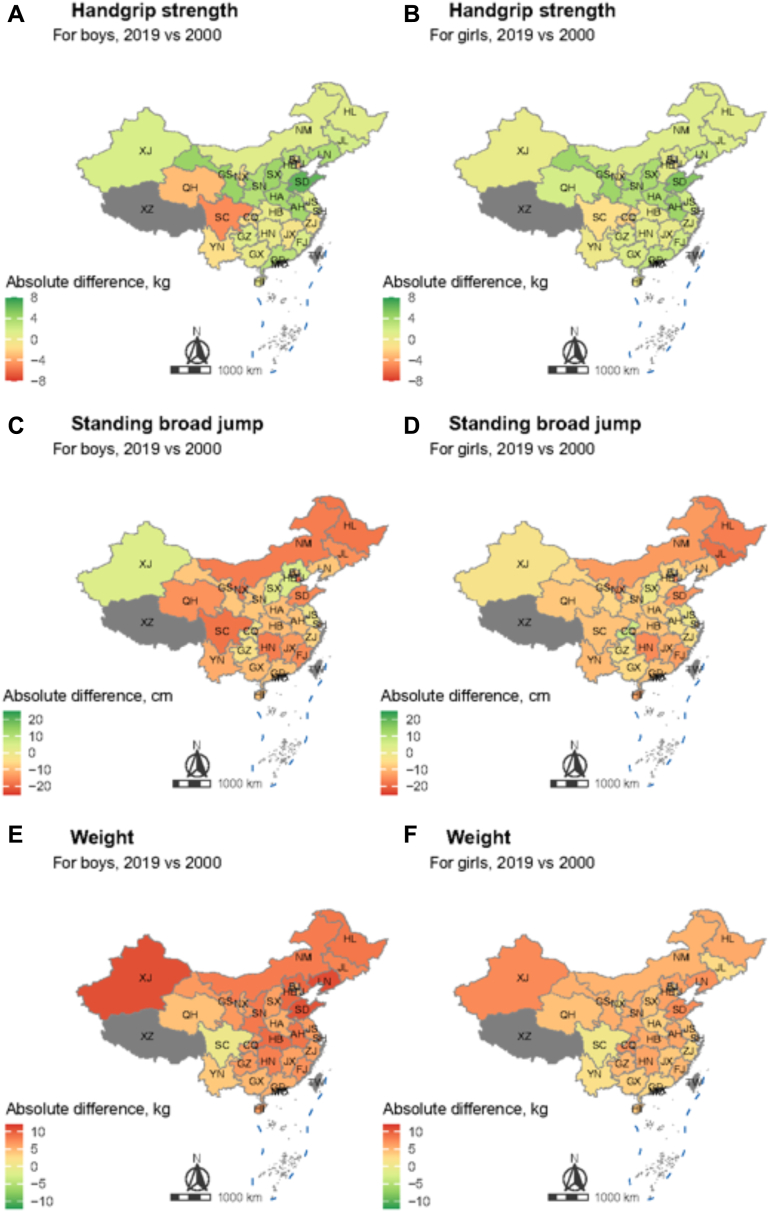
Provincial changes in handgrip strength (A, B), standing broad jump (C, D), and weight (E, F) among students aged 7–22 years by sex, in 2000–2019.

**Fig. 4:**
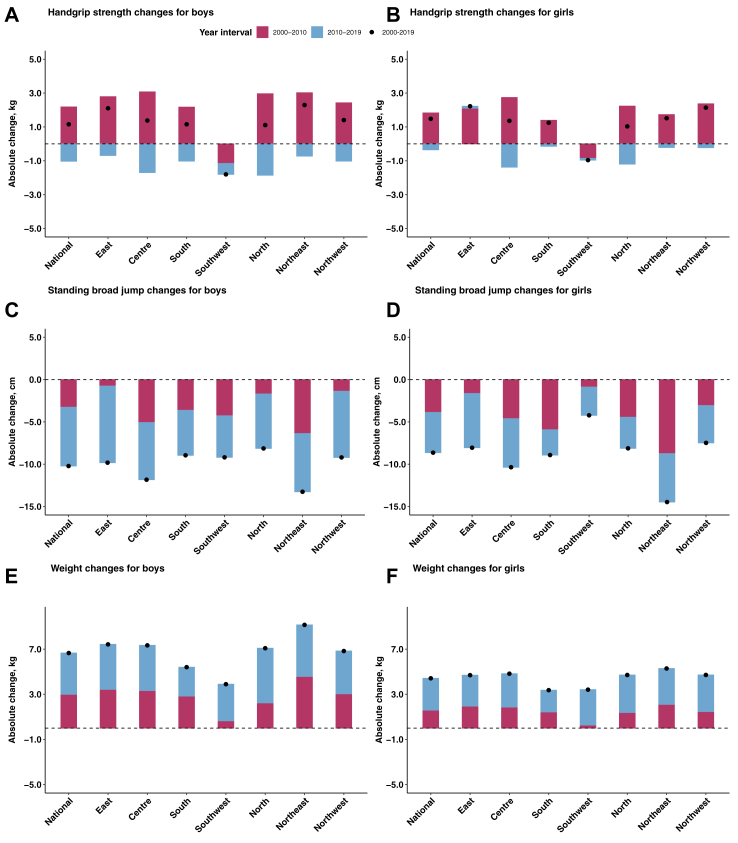
Absolute changes in handgrip strength (A, B), standing broad jump (C, D), and weight (E, F) among students aged 7–22 years by sex and region, in 2000–2019.

(Substantial geographical heterogeneities in absolute changes of handgrip strength, standing broad jump, and body weight were most evident during the most recent decade (2010–2019), as shown in Fig. 3 and Supplementary Fig. S3). Generally, the Southwest region (male: −1.8 kg; female: −1.0 kg) experience continuous downward trend in handgrip strength between 2000 and 2019, and the North (male: −1.9 kg; female: −1.2 kg) and Centre (male: −1.7 kg; female: −1.4 kg) region had above-average (male: −1.0 kg; female: −0.4 kg) decrease during the most recent decade. Female students from the East region (male: 2.1 kg; female: 2.2 kg) had continuous but slight increase during the two decades. Besides, the regional trends of female’ handgrip strength were similar with male (Fig. 4A and B). The Northeast (−13.3 cm for male and −14.5 cm for female) and Centre (−11.8 cm for male and −10.4 cm for female) regions experienced the greatest decline in standing broad jump in both male and female. The decline was significantly more prominent in the most recent decade than in the previous one. Changes in other regions and in female students (except for Southwest region) were generally consistent (Fig. 4C and D). Students’ weight increased significantly during the past two decades in all regions, with continuous growing rates. The highest cumulative weight gain was found in Northeast region for both male (9.2 kg) and female (5.3 kg) (Fig. 4E and F).

### Associations between muscle strength and weight

**Fig. 5:**
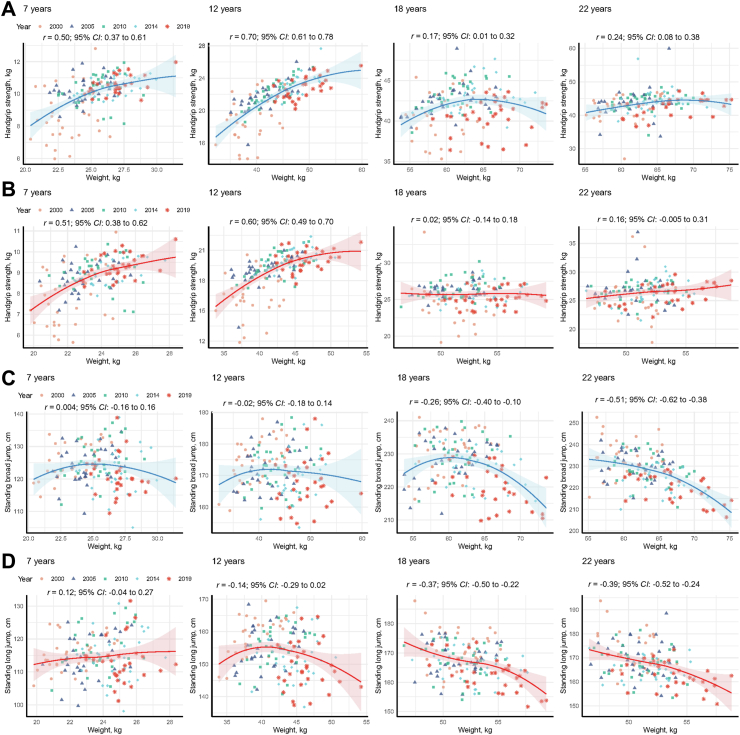
Association between muscle strength and weight among students aged 7–22 years by sex (A and C for boys; B and D for girls) in mainland China, in 2000–2019.

Overall, body weight was positively associated with handgrip strength, and negatively associated with standing broad jump (Supplementary Fig. S4). With advancing age, the strength of the positive association between weight and handgrip strength progressively weakened. Specifically, Pearson’s correlation coefficients declined from 0.70 (95% CI: 0.61 to 0.78; boys) and 0.60 (95% CI: 0.49 to 0.70; girls) at age 12 to 0.24 (95% CI: 0.08 to 0.38; male) and 0.16 (95% CI: −0.005 to 0.31; female) by age 22. Conversely, the association between weight and standing broad jump shifted dynamically across age groups: correlations were initially non-significant at ages 7 and 12 but became inversely significant by age 22, reaching −0.51 (95% CI: −0.62 to −0.38) for male and −0.39 (95% CI: −0.52 to −0.24) for female (Fig. 5).

## Discussion

This study provides a comprehensive analysis of trends in muscle strength and body weight among 1.33 million students from 30 provinces in China, using data from five successive waves of the CNSSCH between 2000 and 2019. Over the past two decades, both upper and lower body muscle strength has shown significant declines unproportioned to weight gain, indicating a growing imbalance. As was discovered in our present study, this mismatch is most pronounced in lower age- and sex-specific strength quantiles, and among the 18- to 22-year-olds, a demographic typically considered to be at the peak of muscle strength development. The most significant declines were observed in Northeast China, North China, and Central China. These findings suggests that incorporating muscle strength training into obesity prevention and control strategies is essential to better promote the overall healthy development of children in these key areas. The concerning divergence in trends of weight and muscle strength development also suggests that increasing body weight is not accompanied by proportional improvements in muscle strength, posing potential risks to the physical health and developmental outcomes of Chinese students. Other research indicates a decline in muscle-strengthening exercise among adolescents with increasing age. In China, according to the 2019 Physical Activity and Fitness in China-The Youth Study, less than two-fifths of Chinese students met the World Health Organization’s recommendations of muscle-strengthening exercise.^31^ International studies also indicate that Asian adolescents tend to have worse fitness test performances levels than other races/ethnicities.^32^^,^^33^ The failure to specify exercise modalities has resulted in physical activity regimens that are frequently inadequate for developing muscle strength. The concurrent rise in adolescent obesity rates alongside declining muscular fitness underscores an urgent need for systematic monitoring and intervention. Without coordinated national efforts, these trends threaten the long-term health development of Chinese youth and have adverse implications for adult health. The 2023 release of the *Hygienic requirements for sport load in physical exercise of primary and middle school students* (Standard No. WS/T 10007-2023) by the National Administration of Disease Control and Prevention represents a pivotal step toward addressing these critical gaps.

Muscle strength is a critical component of physical fitness and has been strongly linked to reduced risks of early death, cardiovascular diseases, and physical disability.^3^ Although the concept of sarcopenic obesity and its related morbidities have not been investigated in detail in the pediatric population, evidence suggests that insufficient muscle mass and strength during childhood and adolescence may foster the development of a sarcopenic-like obesity phenotype, which is characterized by reduced muscular function and strength, despite excessive weight gain.^34^ Importantly, once insufficient peak muscle strength develops, regaining it later in life requires considerably greater effort, emphasizing the importance of monitoring and preserving muscle strength during critical periods of growth and development.^35^, ^36^, ^37^

To our knowledge, this is the very first study that analyses changes in muscle strength in Chinese students over the last 20 years using nationally representative data. Building on previous multi-country studies that included China,^18^^,^^19^^,^^38^ our findings highlight a continued decline in muscle strength, particularly in the most recent survey waves. For handgrip strength, boys experienced a slowing growth rate between 2000 and 2014, followed by a significant decline after 2014, especially at ages 18–22. Among girls, the decline in handgrip strength began in 2010, with the sharpest decreases also observed in the 18- to 22-year-old group. While many provinces showed overall improvements in handgrip strength between 2000 and 2019, the rate of decline has accelerated in recent years, with 30% of provinces for boys (9 of 30) and 20% of provinces for girls (6 of 30) falling below their 2000 levels. Standing broad jump exhibited an even more consistent decline. Slight improvements were observed in specific age groups (e.g., boys aged 7, 12, and 18 and girls aged 7) during the 2005–2010 wave, but most age groups and survey waves showed continued decreases. By the most recent survey, only three provinces for boys and one province for girls exceeded their 2000 standing broad jump levels. The majority of provinces with the most significant muscle strengths declines are classified as regions with a high prevalence of child and adolescent obesity, according to the National Health Committee of the People’s Republic of China.^39^ Concurrently, insufficient dietary diversity, failure to meet physical activity guidelines, and inadequate muscle-strengthening exercise among students in certain regions may all contribute to these disparities.^40^ The most pronounced declines in muscle strength (particularly in lower body) were observed in Northeast China, Shandong, and several inland provinces; the specific regional drivers underlying this pattern warrant further investigation.

The persistent downward shift in the distribution of both upper and lower body muscle strength suggests that this decline will likely worsen without targeted interventions. These findings aligned with previous findings from several national studies from Lithuania^41^ and the United Kingdom,^42^ which also reported declining muscular strength among youth. Conversely, evidence from the United States suggested slight improvements in standing broad jump performance during the late 20th century, though the test has since been removed from many national surveillance programs,^43^ making recent comparisons impossible.

Although muscle strength has been widely recognized as a critical indicator of growth and developmental potential as well as long-term health outcomes,^44^^,^^45^ normative data for such indicators, namely handgrip strength and standing broad jump in children and adolescents have yet to be established.^34^ One previous study leveraging data from thousands of children in Hong Kong, have developed distribution curves for these metrics.^46^ However, these findings are limited in scope and are insufficient to represent the broader population of China or the wider Asian region. While global normative data for handgrip strength have been developed using adult samples,^13^ there remains a significant gap in evidence for younger populations, particularly in Asia. The data from this study could serve as a valuable reference for understanding the distribution of muscle strength among key populations in China and neighboring countries. Furthermore, it provides foundational evidence from Asia to support the development of normative standards for muscle strength in children and young adults. Given the strong associations between muscle strength, body weight, and unhealthy lifestyle behaviors,^47^ targeted interventions aimed at improving muscle strength could play an essential role in advancing national health strategies. By integrating muscle strength assessments and interventions into public health frameworks, policymakers and practitioners can better address the dual challenges of rising childhood obesity and declining physical fitness, ultimately fostering healthier generations and achieving long-term public health goals.

This study has several strengths. The large nationally representative sample size, covering 1.33 million participants across 30 provinces, provides a panoramic view of declining trends of muscle strength corresponding with increasing weight gain, which indicates a warning signals of cardiometabolic health in their later adulthood. The consistent use of standardized measurement and evaluation methods across five survey waves ensures the reliability and comparability of the results over time. Moreover, these strengths deserve the future relevant research and offer valuable insights into the changing dynamics of muscle strength and body weight in this population.

However, the study has several limitations that should be acknowledged. First, this analysis was limited to school-attending Han Chinese children and young adults, excluding ethnic minorities, out-of-school youth, and populations from Hong Kong, Macao, and Taiwan. This exclusion may affect the generalizability of our findings to certain regions, ethnic groups, and non-school-attending population. According to the latest data (2021) from the National Bureau of Statistics, the Han population accounts for 91.1% of China’s total population, while the 2022 Overview of Education in China by the Ministry of Education reports enrollment rates of >99% in primary and junior high schools, >95% in senior high schools, and 60% in universities.^48^ Therefore, the national representativeness of this study should be confined to the student population (particularly those aged 18–22 years) and cannot be generalized to non-student groups. Second, the study is based on multiple cross-sectional surveys, which allow for the analysis of associations but not causal relationships between muscle strength and body weight. Longitudinal studies are needed to elucidate the causal mechanisms underlying these associations and to better understand the long-term implications on population of these trends. Further research on the underlying factors contributing to the observed trends, including lifestyle, nutrition, and environmental influences affecting muscle strength and weight gain are in need. Also, the effects of reduced muscle strength on adolescent growth potential and long-term health are not known and requires more follow-up studies. Third, while handgrip strength and standing broad jump are widely regarded as key indicators for muscle strength for upper and lower limbs, respectively, they do not fully reflect muscle strength in other parts of the body, such as the trunk. In addition, these strength measures do not necessarily correlate with muscle mass. Future research may consider exploring the relationship between body weight and muscle strength in a more comprehensive manner, encompassing other muscle groups.

In conclusion, this national-representative study reveals a worrying declining in muscles strength that is inversely correlated with body weight among Chinese students over the past two decades. The steepest declines occurred in the 18- to 22-year-old age group, a critical period for attaining peak muscle strength, and in North and Northeast China, where obesity was highly prevalent, implying the heavier disease burden in the future. These findings demonstrate a mismatch between increasing body weight and declining muscle strength, and highlight the need for region-specific interventions to increase peak muscle strength to maximize their long-term health expectancy in young population.

## Contributors

YS and ZZ conceptualised the study and contributed to the study design. XW, HW, and XY conducted the data extraction and curation, investigation, methodology, validation, formal analyses, visualisation, and writing of the original draft. YH and RSS contributed to the study design, data extraction, data curation, investigation, formal analysis, investigation, visualisation, and writing. SC contributed to the investigation, data extraction and curation. XW, YS, and ZZ contributed to the funding acquisition. XW, HW, XY, YS, and ZZ accessed and verified the data reported in the paper. All authors reviewed and edited the manuscript, approved the final version, and had final responsibility for the decision to submit for publication.

## Data sharing statement

All data in this article can be shared. Requests with appropriate ethics board approvals and study protocols will be assessed by the Institute of Child and Adolescent Health, Peking University.

## Editor note

The Lancet Group takes a neutral position with respect to territorial claims in published maps and institutional affiliations.

## Declaration of interests

The authors declare no competing interests.
