# Supplementary material for: Imbalance between muscle strength development and weight gain in children and young adults in China: serial cross-sectional evidence from 1.33 million students from five successive national surveys between 2000 and 2019
Source: Lancet Reg Health West Pac. 2025 Jul 25;61:101640. doi: 10.1016/j.lanwpc.2025.101640 (PMC12311536; doi:10.1016/j.lanwpc.2025.101640)
Supplement: Supplementary Figs. S1–S4 and Tables S1–S3 [file mmc1.docx]

**Supplementary materials**

- Table S1: Muscle strength and weight in 30 provinces of the Chinese National Survey on Students’ Constitution and Health, in 2000–2019.
- Table S2: Handgrip strength (kg)/height^2^ (m^2^) by age, sex, and region of the Chinese National Survey on Students’ Constitution and Health, in 2000–2019.
- Table S3: Differences in change of muscle strength and weight by age and sex of the Chinese National Survey on Students’ Constitution and Health, in 2000–2010, 2010–2019
- Figure S1: Temporal trends in handgrip strength (A, B), standing broad jump (C, D), and weight (E, F) by age and sex in mainland China, in 2000–2019.
- Figure S2: Absolute changes in handgrip strength (A, B), standing broad jump (C, D), and weight (E, F) by age and sex in mainland China, in 2000–2019.
- Figure S3: Provincial changes in handgrip strength (A, B), standing broad jump (C, D), and weight (E, F) among students aged 7–22 years by sex, in 2010–2019.
- Figure S4: Association between muscle strength and weight among students aged 7–22 years by sex (A and C for boys; B and D for girls) in mainland China, in 2000–2019.

# Table S1: Muscle strength and weight in 30 provinces of the Chinese National Survey on Students’ Constitution and Health, in 2000–2019

|  | **2000** | |  | **2005** | |  | **2010** | |  | **2014** | |  | **2019** | |
| --- | --- | --- | --- | --- | --- | --- | --- | --- | --- | --- | --- | --- | --- | --- |
|  | **Boys** | **Girls** |  | **Boys** | **Girls** |  | **Boys** | **Girls** |  | **Boys** | **Girls** |  | **Boys** | **Girls** |
| **Sample size** | **133,742** | **132,548** |  | **146,213** | **145,391** |  | **131,457** | **131,421** |  | **130,944** | **130,970** |  | **128,438** | **127,936** |
| **Handgrip strength, kg** |  |  |  |  |  |  |  |  |  |  |  |  |  |  |
| Overall | 27.3 (27.3~27.4) | 18.7 (18.7~18.8) |  | 29.4 (29.4~29.5) | 20.5 (20.5~20.5) |  | 29.5 (29.5~29.6) | 20.6 (20.5~20.6) |  | 29.6 (29.6~29.7) | 20.6 (20.5~20.6) |  | 28.5 (28.4~28.6) | 20.2 (20.2~20.3) |
| Liaoning | 24.8 (24.5, 25.2) | 18.0 (17.8, 18.1) |  | 30.9 (30.5, 31.3) | 21.0 (20.8, 21.2) |  | 30.2 (29.8, 30.6) | 20.9 (20.7, 21.1) |  | 29.9 (29.5, 30.3) | 20.5 (20.3, 20.7) |  | 28.3 (27.9, 28.7) | 20.0 (19.8, 20.2) |
| Xinjiang | 27.0 (26.2, 27.7) | 19.7 (19.3, 20.1) |  | 29.7 (29.3, 30.1) | 19.9 (19.7, 20.1) |  | 28.5 (28.1, 28.9) | 20.0 (19.8, 20.3) |  | 29.4 (29.0, 29.8) | 20.1 (19.9, 20.4) |  | 28.6 (28.2, 29.0) | 20.1 (19.9, 20.3) |
| Shandong | 25.3 (25.0, 25.7) | 16.9 (16.7, 17.0) |  | 29.7 (29.3, 30.1) | 20.3 (20.1, 20.6) |  | 31.0 (30.5, 31.4) | 20.8 (20.6, 21.0) |  | 31.0 (30.5, 31.4) | 21.2 (21.0, 21.4) |  | 31.7 (31.3, 32.1) | 22.0 (21.8, 22.2) |
| Hebei | 27.2 (26.8, 27.6) | 20.2 (20.0, 20.5) |  | 33.8 (33.5, 34.2) | 22.6 (22.4, 22.8) |  | 31.3 (30.7, 31.9) | 22.5 (22.1, 22.8) |  | 32.3 (31.8, 32.8) | 22.4 (22.2, 22.7) |  | 30.2 (29.7, 30.6) | 20.8 (20.5, 21.0) |
| Hubei | 28.3 (27.9, 28.7) | 19.6 (19.4, 19.9) |  | 28.9 (28.4, 29.5) | 20.7 (20.4, 21.0) |  | 30.6 (30.2, 31.0) | 21.7 (21.5, 22.0) |  | 30.5 (30.1, 30.9) | 21.4 (21.2, 21.6) |  | 28.7 (28.3, 29.1) | 19.8 (19.6, 20.1) |
| Anhui | 26.1 (25.6, 26.5) | 17.1 (16.9, 17.4) |  | 29.5 (29.1, 29.9) | 20.8 (20.6, 21.1) |  | 30.8 (30.4, 31.2) | 21.8 (21.5, 22.0) |  | 30.0 (29.6, 30.4) | 21.1 (20.9, 21.3) |  | 30.0 (29.6, 30.4) | 21.3 (21.0, 21.5) |
| Heilongjiang | 28.6 (28.1, 29.0) | 20.0 (19.8, 20.2) |  | 28.0 (27.6, 28.3) | 19.7 (19.5, 19.9) |  | 29.6 (29.1, 30.0) | 20.2 (19.9, 20.4) |  | 29.0 (28.6, 29.4) | 20.7 (20.4, 20.9) |  | 29.5 (29.1, 29.9) | 20.8 (20.6, 21.1) |
| Shaanxi | 25.1 (24.7, 25.5) | 16.4 (16.2, 16.6) |  | 29.4 (29.0, 29.8) | 20.6 (20.4, 20.9) |  | 29.2 (28.8, 29.6) | 20.5 (20.3, 20.7) |  | 29.3 (28.9, 29.7) | 20.3 (20.0, 20.5) |  | 28.3 (27.9, 28.7) | 19.4 (19.2, 19.6) |
| Inner Mongolia | 29.0 (28.6, 29.4) | 20.0 (19.8, 20.3) |  | 29.1 (28.7, 29.5) | 20.0 (19.8, 20.2) |  | 31.1 (30.7, 31.6) | 21.5 (21.3, 21.8) |  | 31.6 (31.1, 32.0) | 22.0 (21.7, 22.2) |  | 30.0 (29.6, 30.4) | 21.2 (21.0, 21.5) |
| Hunan | 27.2 (26.8, 27.6) | 19.2 (18.9, 19.4) |  | 28.3 (27.9, 28.7) | 20.2 (19.9, 20.4) |  | 29.8 (29.4, 30.2) | 20.9 (20.7, 21.1) |  | 31.3 (30.7, 31.8) | 19.4 (19.2, 19.7) |  | 27.4 (27.0, 27.8) | 19.8 (19.6, 20.0) |
| Jilin | 26.7 (26.3, 27.1) | 18.7 (18.5, 18.9) |  | 31.9 (31.5, 32.3) | 21.6 (21.3, 21.8) |  | 28.7 (28.3, 29.1) | 20.4 (20.2, 20.7) |  | 30.9 (30.5, 31.4) | 21.2 (21.0, 21.5) |  | 28.4 (28.0, 28.9) | 19.9 (19.7, 20.2) |
| Shanghai | 25.0 (24.7, 25.4) | 18.1 (17.9, 18.3) |  | 29.9 (29.5, 30.3) | 20.3 (20.1, 20.5) |  | 29.2 (28.8, 29.6) | 19.7 (19.5, 19.9) |  | 28.8 (28.4, 29.2) | 20.5 (20.3, 20.7) |  | 26.6 (26.2, 27.0) | 19.2 (19.0, 19.4) |
| Fujian | 26.9 (26.5, 27.4) | 18.2 (18.0, 18.5) |  | 30.1 (29.7, 30.5) | 20.7 (20.5, 20.9) |  | 28.2 (27.9, 28.6) | 20.0 (19.8, 20.2) |  | 28.5 (28.1, 28.9) | 20.0 (19.9, 20.2) |  | 28.4 (28.0, 28.8) | 20.0 (19.8, 20.3) |
| Gansu | 26.1 (25.8, 26.5) | 16.6 (16.3, 16.8) |  | 29.1 (28.8, 29.5) | 20.6 (20.4, 20.8) |  | 29.7 (29.2, 30.1) | 20.2 (20.0, 20.4) |  | 30.1 (29.7, 30.6) | 20.3 (20.0, 20.5) |  | 30.5 (30.1, 30.9) | 20.7 (20.5, 20.9) |
| Guizhou | 26.2 (25.8, 26.6) | 18.5 (18.3, 18.7) |  | 27.6 (27.2, 28.0) | 19.9 (19.7, 20.2) |  | 27.7 (27.4, 28.1) | 20.1 (19.9, 20.4) |  | 27.8 (27.4, 28.2) | 19.9 (19.7, 20.1) |  | 27.3 (26.9, 27.7) | 19.5 (19.3, 19.7) |
| Hainan | 26.9 (26.6, 27.3) | 20.0 (19.8, 20.3) |  | 26.1 (25.7, 26.5) | 18.1 (17.8, 18.3) |  | 27.7 (27.3, 28.1) | 19.8 (19.6, 20.1) |  | 27.7 (27.3, 28.1) | 19.9 (19.7, 20.1) |  | 27.3 (26.9, 27.7) | 19.9 (19.7, 20.2) |
| Chongqing | 29.4 (29.0, 29.9) | 24.4 (24.0, 24.8) |  | 25.5 (25.2, 25.8) | 18.5 (18.3, 18.6) |  | 27.5 (27.1, 27.9) | 20.0 (19.8, 20.2) |  | 28.1 (27.7, 28.4) | 19.7 (19.5, 19.9) |  | 27.9 (27.5, 28.3) | 22.2 (22.0, 22.4) |
| Jiangsu | 28.0 (27.5, 28.4) | 18.6 (18.3, 18.8) |  | 30.9 (30.5, 31.2) | 21.1 (20.9, 21.3) |  | 29.9 (29.4, 30.3) | 20.2 (19.9, 20.4) |  | 30.6 (30.2, 31.1) | 21.1 (20.9, 21.3) |  | 29.2 (28.8, 29.6) | 20.5 (20.3, 20.7) |
| Jiangxi | 29.6 (29.2, 30.0) | 20.1 (19.9, 20.3) |  | 28.1 (27.7, 28.6) | 19.8 (19.6, 20.0) |  | 29.2 (28.8, 29.6) | 20.3 (20.1, 20.5) |  | 29.3 (28.9, 29.8) | 20.1 (19.8, 20.3) |  | 28.8 (28.4, 29.2) | 20.3 (20.1, 20.6) |
| Shanxi | 24.8 (24.4, 25.2) | 16.5 (16.2, 16.7) |  | 29.9 (29.5, 30.3) | 21.1 (20.8, 21.3) |  | 31.1 (30.4, 31.7) | 21.8 (21.5, 22.1) |  | 30.4 (30.0, 30.8) | 21.4 (21.2, 21.6) |  | 28.9 (28.5, 29.3) | 20.1 (19.9, 20.3) |
| Zhejiang | 28.5 (28.1, 29.0) | 18.7 (18.5, 18.9) |  | 30.2 (29.8, 30.6) | 21.3 (21.1, 21.6) |  | 29.3 (28.8, 29.7) | 19.6 (19.3, 19.8) |  | 28.1 (27.7, 28.5) | 19.4 (19.1, 19.6) |  | 27.7 (27.3, 28.0) | 19.5 (19.3, 19.7) |
| Tianjin | 31.7 (31.2, 32.2) | 21.0 (20.7, 21.3) |  | 30.7 (30.3, 31.0) | 20.4 (20.2, 20.6) |  | 31.1 (30.5, 31.7) | 21.3 (21.0, 21.6) |  | 30.6 (30.2, 31.0) | 20.8 (20.6, 21.0) |  | 28.9 (28.5, 29.3) | 19.9 (19.7, 20.1) |
| Beijing | 29.5 (29.1, 29.9) | 20.2 (20.0, 20.4) |  | 31.1 (30.7, 31.5) | 21.3 (21.0, 21.5) |  | 31.9 (31.3, 32.4) | 21.7 (21.4, 22.0) |  | 29.2 (28.8, 29.7) | 20.4 (20.2, 20.6) |  | 29.1 (28.7, 29.5) | 20.6 (20.3, 20.8) |
| Ningxia | 28.5 (28.1, 29.0) | 20.0 (19.7, 20.2) |  | 30.2 (29.8, 30.6) | 21.5 (21.2, 21.7) |  | 30.5 (30.1, 31.0) | 21.2 (21.0, 21.5) |  | 31.0 (30.5, 31.4) | 21.7 (21.4, 21.9) |  | 27.1 (26.7, 27.6) | 19.8 (19.5, 20.1) |
| Guangdong | 25.2 (24.8, 25.6) | 17.7 (17.5, 17.9) |  | 28.9 (28.5, 29.3) | 20.7 (20.5, 21.0) |  | 28.8 (28.4, 29.2) | 19.9 (19.6, 20.1) |  | 28.7 (28.3, 29.1) | 19.8 (19.6, 20.0) |  | 28.0 (27.7, 28.4) | 20.3 (20.1, 20.5) |
| Henan | 26.6 (26.2, 27.0) | 17.4 (17.2, 17.6) |  | 29.4 (29.0, 29.8) | 20.9 (20.7, 21.1) |  | 30.9 (30.5, 31.3) | 21.7 (21.5, 22.0) |  | 31.9 (31.5, 32.4) | 22.5 (22.2, 22.7) |  | 30.3 (29.9, 30.7) | 20.7 (20.5, 20.9) |
| Guangxi | 27.0 (26.5, 27.4) | 18.7 (18.4, 18.9) |  | 28.0 (27.6, 28.5) | 19.8 (19.6, 20.1) |  | 29.2 (28.8, 29.6) | 20.8 (20.6, 21.1) |  | 28.7 (28.3, 29.1) | 20.5 (20.2, 20.7) |  | 27.2 (26.8, 27.6) | 19.8 (19.6, 20.1) |
| Qinghai | 28.5 (27.8, 29.2) | 18.5 (18.2, 18.9) |  | 27.8 (27.3, 28.2) | 19.8 (19.6, 20.1) |  | 28.3 (27.9, 28.7) | 19.4 (19.1, 19.6) |  | 28.4 (28.0, 28.8) | 20.1 (19.9, 20.3) |  | 25.5 (25.0, 26.0) | 20.3 (19.9, 20.6) |
| Yunnan | 28.9 (28.4, 29.5) | 19.3 (19.1, 19.6) |  | 28.5 (28.1, 28.9) | 19.9 (19.7, 20.2) |  | 29.1 (28.7, 29.6) | 20.6 (20.4, 20.8) |  | 28.0 (27.6, 28.4) | 19.2 (19.0, 19.4) |  | 27.4 (27.0, 27.7) | 19.1 (18.9, 19.3) |
| Sichuan | 31.2 (30.9, 31.6) | 20.7 (20.4, 20.9) |  | 30.7 (30.3, 31.0) | 21.6 (21.4, 21.7) |  | 27.2 (26.7, 27.6) | 19.1 (18.8, 19.3) |  | 28.0 (27.6, 28.4) | 19.5 (19.3, 19.7) |  | 26.4 (26.0, 26.8) | 19.0 (18.8, 19.2) |
| **Standing broad jump, cm** |  |  |  |  |  |  |  |  |  |  |  |  |  |  |
| Overall | 193.3 (193.1~193.5) | 157.8 (157.7~158.0) |  | 190.2 (190.0~190.5) | 154.3 (154.2~154.5) |  | 190.0 (189.8~190.3) | 154.0 (153.8~154.1) |  | 186.2 (186.0~186.4) | 152.0 (151.9~152.2) |  | 183.1 (182.9~183.3) | 149.2 (149.1~149.3) |
| Liaoning | 188.6 (187.6, 189.7) | 156.9 (156.3, 157.5) |  | 196.0 (194.8, 197.1) | 156.8 (156.1, 157.4) |  | 193.4 (192.1, 194.7) | 156.5 (155.8, 157.1) |  | 188.2 (187.0, 189.5) | 154.8 (154.1, 155.5) |  | 180.5 (179.2, 181.8) | 148.3 (147.5, 149.1) |
| Xinjiang | 183.0 (180.6, 185.4) | 154.9 (153.5, 156.4) |  | 186.3 (185.2, 187.3) | 153.2 (152.6, 153.8) |  | 188.2 (186.8, 189.5) | 153.4 (152.6, 154.1) |  | 182.9 (181.6, 184.3) | 149.5 (148.7, 150.3) |  | 187.1 (185.7, 188.4) | 152.1 (151.4, 152.9) |
| Shandong | 196.7 (195.6, 197.8) | 159.5 (158.8, 160.1) |  | 191.3 (190.2, 192.5) | 155.6 (154.9, 156.3) |  | 191.3 (190.1, 192.5) | 155.2 (154.4, 155.9) |  | 186.5 (185.2, 187.8) | 153.3 (152.5, 154.1) |  | 181.3 (180.1, 182.5) | 145.5 (144.8, 146.2) |
| Hebei | 184.3 (182.9, 185.8) | 156.8 (155.9, 157.6) |  | 192.8 (191.7, 193.8) | 150.2 (149.5, 150.8) |  | 188.3 (186.4, 190.2) | 148.7 (147.6, 149.8) |  | 185.3 (183.8, 186.7) | 149.1 (148.3, 149.9) |  | 188.4 (187.0, 189.7) | 150.2 (149.5, 151.0) |
| Hubei | 190.7 (189.4, 191.9) | 155.8 (155.1, 156.5) |  | 193.1 (191.5, 194.6) | 157.8 (157.0, 158.7) |  | 189.0 (187.8, 190.3) | 152.9 (152.2, 153.6) |  | 188.3 (187.1, 189.6) | 153.8 (153.1, 154.5) |  | 181.4 (180.2, 182.6) | 147.2 (146.5, 148.0) |
| Anhui | 189.9 (188.6, 191.1) | 152.5 (151.8, 153.3) |  | 188.4 (187.2, 189.7) | 153.0 (152.3, 153.7) |  | 187.9 (186.6, 189.1) | 151.0 (150.3, 151.7) |  | 186.7 (185.4, 188.1) | 152.5 (151.7, 153.3) |  | 181.3 (180.0, 182.6) | 148.4 (147.6, 149.1) |
| Heilongjiang | 192.4 (191.0, 193.7) | 157.1 (156.2, 158.0) |  | 188.7 (187.4, 190.1) | 153.7 (152.8, 154.6) |  | 180.0 (178.6, 181.3) | 141.9 (141.2, 142.7) |  | 177.2 (175.7, 178.6) | 143.0 (142.0, 143.9) |  | 174.7 (173.4, 176.0) | 140.4 (139.6, 141.2) |
| Shaanxi | 185.0 (183.8, 186.2) | 150.7 (150.0, 151.4) |  | 187.2 (186.1, 188.4) | 152.0 (151.4, 152.7) |  | 183.3 (182.1, 184.5) | 146.6 (145.9, 147.3) |  | 179.4 (178.2, 180.7) | 144.1 (143.3, 144.9) |  | 176.6 (175.4, 177.9) | 142.3 (141.6, 143.0) |
| Inner Mongolia | 195.8 (194.5, 197.1) | 158.5 (157.7, 159.3) |  | 191.4 (190.2, 192.6) | 155.1 (154.4, 155.8) |  | 187.8 (186.5, 189.1) | 151.9 (151.2, 152.7) |  | 184.2 (182.9, 185.5) | 148.0 (147.2, 148.7) |  | 178.9 (177.6, 180.2) | 145.1 (144.3, 145.9) |
| Hunan | 199.5 (198.3, 200.7) | 165.1 (164.4, 165.8) |  | 196.2 (195.0, 197.3) | 161.5 (160.8, 162.1) |  | 190.7 (189.5, 191.8) | 157.0 (156.4, 157.7) |  | 187.8 (186.6, 189.0) | 155.2 (154.5, 156.0) |  | 182.6 (181.4, 183.8) | 150.0 (149.3, 150.8) |
| Jilin | 191.2 (189.8, 192.5) | 157.9 (157.0, 158.8) |  | 186.0 (184.8, 187.2) | 148.0 (147.3, 148.7) |  | 178.8 (177.5, 180.1) | 147.1 (146.3, 148.0) |  | 180.7 (179.4, 182.0) | 146.0 (145.3, 146.8) |  | 176.4 (174.9, 177.8) | 139.5 (138.6, 140.3) |
| Shanghai | 194.0 (193.1, 194.9) | 160.8 (160.3, 161.3) |  | 196.0 (194.8, 197.2) | 158.1 (157.5, 158.8) |  | 194.7 (193.5, 195.9) | 157.2 (156.6, 157.8) |  | 187.6 (186.4, 188.9) | 153.9 (153.2, 154.6) |  | 184.7 (183.5, 186.0) | 153.4 (152.7, 154.2) |
| Fujian | 196.5 (195.3, 197.7) | 161.0 (160.3, 161.8) |  | 202.1 (200.9, 203.2) | 162.8 (162.2, 163.5) |  | 200.5 (199.3, 201.8) | 163.3 (162.6, 164.0) |  | 191.9 (190.7, 193.1) | 157.6 (156.9, 158.3) |  | 181.4 (180.1, 182.6) | 147.9 (147.2, 148.6) |
| Gansu | 188.3 (187.2, 189.3) | 152.2 (151.5, 152.8) |  | 179.5 (178.5, 180.5) | 147.8 (147.2, 148.4) |  | 184.2 (183.1, 185.4) | 148.8 (148.1, 149.5) |  | 179.8 (178.5, 181.0) | 145.2 (144.5, 145.9) |  | 180.3 (179.0, 181.6) | 146.1 (145.3, 146.8) |
| Guizhou | 182.6 (181.5, 183.7) | 149.1 (148.4, 149.8) |  | 181.5 (180.3, 182.7) | 147.7 (147.1, 148.4) |  | 182.0 (180.8, 183.1) | 148.5 (147.8, 149.1) |  | 184.7 (183.5, 185.9) | 151.5 (150.8, 152.1) |  | 178.6 (177.3, 179.9) | 145.4 (144.6, 146.1) |
| Hainan | 195.7 (194.2, 197.2) | 162.5 (161.5, 163.4) |  | 184.6 (183.6, 185.7) | 149.8 (149.2, 150.5) |  | 193.2 (192.0, 194.4) | 157.5 (156.8, 158.2) |  | 185.2 (184.0, 186.4) | 151.6 (150.9, 152.4) |  | 186.7 (185.5, 188.0) | 150.3 (149.5, 151.1) |
| Chongqing | 188.9 (187.7, 190.2) | 152.6 (151.9, 153.3) |  | 185.5 (184.4, 186.7) | 153.2 (152.5, 153.9) |  | 194.6 (193.4, 195.8) | 160.7 (160.1, 161.4) |  | 192.2 (191.0, 193.4) | 158.4 (157.7, 159.0) |  | 186.9 (185.6, 188.3) | 158.7 (157.9, 159.5) |
| Jiangsu | 197.2 (196.0, 198.4) | 159.2 (158.5, 159.9) |  | 198.2 (197.2, 199.2) | 157.5 (157.0, 158.0) |  | 197.6 (196.4, 198.8) | 160.0 (159.4, 160.7) |  | 194.4 (193.2, 195.6) | 158.3 (157.6, 159.0) |  | 195.6 (194.4, 196.8) | 158.4 (157.6, 159.1) |
| Jiangxi | 197.2 (196.0, 198.4) | 160.1 (159.4, 160.8) |  | 188.4 (187.2, 189.7) | 155.5 (154.8, 156.3) |  | 195.6 (194.3, 196.8) | 158.4 (157.7, 159.1) |  | 185.3 (184.0, 186.6) | 151.7 (150.9, 152.5) |  | 183.8 (182.4, 185.1) | 149.3 (148.6, 150.1) |
| Shanxi | 185.7 (184.5, 186.9) | 150.8 (150.0, 151.6) |  | 186.5 (185.2, 187.8) | 148.6 (147.8, 149.4) |  | 188.9 (187.1, 190.8) | 149.9 (148.9, 151.0) |  | 188.3 (187.0, 189.6) | 152.0 (151.3, 152.8) |  | 187.0 (185.6, 188.4) | 150.5 (149.7, 151.3) |
| Zhejiang | 203.3 (202.0, 204.5) | 166.7 (165.9, 167.4) |  | 198.2 (197.0, 199.5) | 162.1 (161.3, 162.8) |  | 200.5 (199.3, 201.7) | 164.2 (163.5, 164.9) |  | 198.2 (197.1, 199.4) | 164.3 (163.6, 164.9) |  | 196.9 (195.9, 198.0) | 162.3 (161.7, 162.9) |
| Tianjin | 197.0 (195.5, 198.4) | 158.9 (158.0, 159.7) |  | 191.2 (190.0, 192.4) | 153.1 (152.5, 153.8) |  | 193.1 (191.3, 194.8) | 155.4 (154.4, 156.4) |  | 187.3 (186.1, 188.5) | 154.7 (154.0, 155.4) |  | 176.5 (175.3, 177.8) | 143.0 (142.3, 143.8) |
| Beijing | 194.8 (193.6, 196.0) | 161.1 (160.4, 161.8) |  | 193.8 (192.6, 195.1) | 159.9 (159.2, 160.6) |  | 192.7 (191.1, 194.3) | 157.8 (156.9, 158.7) |  | 188.4 (187.2, 189.7) | 158.1 (157.5, 158.8) |  | 186.3 (185.2, 187.5) | 155.9 (155.3, 156.6) |
| Ningxia | 188.5 (187.3, 189.8) | 154.0 (153.3, 154.7) |  | 186.4 (185.1, 187.6) | 150.2 (149.5, 151.0) |  | 188.1 (186.8, 189.4) | 151.5 (150.7, 152.2) |  | 178.3 (177.0, 179.6) | 144.7 (144.0, 145.5) |  | 171.2 (169.6, 172.8) | 140.0 (139.0, 141.1) |
| Guangdong | 198.1 (196.8, 199.4) | 164.3 (163.5, 165.0) |  | 193.4 (192.2, 194.7) | 158.2 (157.4, 158.9) |  | 194.4 (193.1, 195.7) | 154.9 (154.2, 155.7) |  | 191.1 (189.7, 192.4) | 154.8 (154.0, 155.6) |  | 189.6 (188.2, 190.9) | 155.5 (154.8, 156.3) |
| Henan | 193.4 (192.2, 194.6) | 154.0 (153.3, 154.7) |  | 185.1 (183.9, 186.2) | 150.7 (150.0, 151.4) |  | 188.8 (187.6, 190.0) | 151.2 (150.5, 151.9) |  | 187.1 (185.8, 188.4) | 151.8 (151.0, 152.5) |  | 184.4 (183.1, 185.8) | 146.4 (145.6, 147.2) |
| Guangxi | 197.2 (196.0, 198.5) | 162.5 (161.8, 163.2) |  | 195.7 (194.4, 196.9) | 161.9 (161.2, 162.6) |  | 192.6 (191.3, 193.9) | 159.1 (158.4, 159.8) |  | 187.8 (186.5, 189.1) | 154.0 (153.3, 154.8) |  | 187.9 (186.5, 189.2) | 156.6 (155.8, 157.4) |
| Qinghai | 182.7 (180.7, 184.7) | 149.3 (148.1, 150.4) |  | 180.1 (178.9, 181.4) | 146.4 (145.6, 147.1) |  | 182.3 (181.0, 183.7) | 145.4 (144.6, 146.1) |  | 177.4 (176.2, 178.7) | 145.5 (144.8, 146.2) |  | 168.3 (166.7, 170.0) | 141.3 (140.4, 142.3) |
| Yunnan | 198.8 (197.3, 200.2) | 160.9 (160.1, 161.7) |  | 191.7 (190.5, 192.9) | 156.3 (155.6, 157.0) |  | 192.2 (190.9, 193.5) | 155.1 (154.4, 155.9) |  | 187.3 (186.0, 188.5) | 151.1 (150.4, 151.8) |  | 187.4 (186.2, 188.7) | 151.3 (150.6, 152.0) |
| Sichuan | 200.2 (199.2, 201.2) | 158.8 (158.1, 159.4) |  | 193.4 (192.5, 194.4) | 156.3 (155.8, 156.9) |  | 186.3 (185.0, 187.5) | 153.2 (152.5, 153.9) |  | 185.8 (184.5, 187.1) | 152.0 (151.2, 152.7) |  | 182.2 (181.0, 183.4) | 150.1 (149.4, 150.8) |
| **Weight, kg** |  |  |  |  |  |  |  |  |  |  |  |  |  |  |
| Overall | 45.7 (45.6~45.8) | 41.4 (41.4~41.5) |  | 47.3 (47.3~47.4) | 42.4 (42.4~42.5) |  | 48.7 (48.6~48.8) | 43.0 (43.0~43.1) |  | 50.6 (50.5~50.7) | 44.4 (44.3~44.5) |  | 52.4 (52.3~52.5) | 45.8 (45.8~45.9) |
| Liaoning | 45.6 (45.2, 46.0) | 41.5 (41.2, 41.8) |  | 52.0 (51.5, 52.4) | 45.6 (45.3, 45.9) |  | 51.4 (50.9, 51.9) | 45.2 (44.8, 45.6) |  | 54.3 (53.7, 54.9) | 47.1 (46.7, 47.5) |  | 56.4 (55.8, 57.0) | 48.4 (48.0, 48.9) |
| Xinjiang | 42.8 (42.0, 43.7) | 39.2 (38.6, 39.9) |  | 47.0 (46.6, 47.4) | 41.8 (41.5, 42.2) |  | 49.2 (48.7, 49.7) | 42.9 (42.6, 43.3) |  | 52.7 (52.2, 53.2) | 45.1 (44.7, 45.5) |  | 53.4 (52.9, 54.0) | 46.6 (46.1, 47.0) |
| Shandong | 50.0 (49.5, 50.5) | 44.1 (43.7, 44.4) |  | 51.5 (51.0, 52.0) | 44.9 (44.5, 45.2) |  | 54.1 (53.5, 54.6) | 46.4 (46.0, 46.8) |  | 55.8 (55.3, 56.4) | 48.4 (48.0, 48.8) |  | 60.4 (59.8, 60.9) | 51.3 (50.9, 51.7) |
| Hebei | 46.3 (45.7, 46.9) | 42.2 (41.7, 42.6) |  | 51.7 (51.2, 52.1) | 45.5 (45.1, 45.8) |  | 49.4 (48.8, 50.1) | 43.4 (42.9, 43.9) |  | 53.6 (53.1, 54.2) | 46.1 (45.7, 46.5) |  | 55.6 (55.1, 56.2) | 48.2 (47.7, 48.6) |
| Hubei | 43.2 (42.7, 43.6) | 39.7 (39.3, 40.1) |  | 45.0 (44.5, 45.6) | 40.7 (40.2, 41.1) |  | 47.6 (47.1, 48.0) | 42.4 (42.0, 42.7) |  | 50.4 (49.9, 50.9) | 43.9 (43.5, 44.3) |  | 52.2 (51.7, 52.8) | 45.2 (44.8, 45.5) |
| Anhui | 44.2 (43.7, 44.6) | 40.2 (39.8, 40.5) |  | 45.0 (44.5, 45.4) | 41.1 (40.8, 41.5) |  | 47.7 (47.3, 48.2) | 42.5 (42.1, 42.8) |  | 50.5 (50.0, 50.9) | 44.2 (43.9, 44.6) |  | 52.8 (52.2, 53.3) | 45.9 (45.5, 46.3) |
| Heilongjiang | 47.9 (47.4, 48.4) | 43.0 (42.6, 43.4) |  | 50.8 (50.3, 51.3) | 44.8 (44.5, 45.2) |  | 51.2 (50.6, 51.7) | 44.7 (44.3, 45.1) |  | 53.3 (52.7, 53.9) | 46.1 (45.7, 46.5) |  | 56.4 (55.8, 57.0) | 48.5 (48.1, 49.0) |
| Shaanxi | 44.1 (43.6, 44.5) | 40.5 (40.1, 40.9) |  | 45.4 (44.9, 45.8) | 41.3 (40.9, 41.7) |  | 47.3 (46.8, 47.8) | 42.2 (41.8, 42.5) |  | 50.6 (50.1, 51.1) | 44.4 (44.1, 44.8) |  | 52.4 (51.9, 52.9) | 45.9 (45.5, 46.3) |
| Inner Mongolia | 46.2 (45.7, 46.7) | 42.1 (41.7, 42.6) |  | 47.6 (47.1, 48.0) | 43.1 (42.8, 43.5) |  | 49.6 (49.1, 50.1) | 44.1 (43.7, 44.5) |  | 52.3 (51.8, 52.8) | 46.0 (45.6, 46.4) |  | 54.3 (53.7, 54.9) | 47.2 (46.8, 47.6) |
| Hunan | 43.9 (43.4, 44.3) | 39.9 (39.5, 40.2) |  | 45.4 (44.9, 45.8) | 40.2 (39.8, 40.5) |  | 47.6 (47.1, 48.1) | 41.8 (41.4, 42.1) |  | 49.3 (48.8, 49.8) | 43.0 (42.7, 43.4) |  | 51.8 (51.3, 52.4) | 45.7 (45.3, 46.1) |
| Jilin | 45.9 (45.4, 46.3) | 44.0 (43.6, 44.3) |  | 49.7 (49.3, 50.2) | 43.3 (42.9, 43.6) |  | 50.2 (49.7, 50.7) | 44.4 (44.0, 44.7) |  | 51.3 (50.9, 51.8) | 46.0 (45.6, 46.3) |  | 53.6 (53.0, 54.2) | 46.7 (46.3, 47.2) |
| Shanghai | 46.5 (46.2, 46.9) | 42.0 (41.7, 42.2) |  | 50.6 (50.1, 51.1) | 44.3 (44.0, 44.7) |  | 52.1 (51.6, 52.6) | 45.1 (44.8, 45.5) |  | 53.4 (52.9, 53.9) | 46.4 (46.0, 46.7) |  | 54.2 (53.6, 54.7) | 46.7 (46.3, 47.1) |
| Fujian | 45.8 (45.3, 46.2) | 40.4 (40.1, 40.8) |  | 48.2 (47.8, 48.6) | 42.8 (42.5, 43.1) |  | 48.7 (48.2, 49.2) | 42.7 (42.4, 43.1) |  | 49.9 (49.4, 50.4) | 43.7 (43.4, 44.1) |  | 52.7 (52.2, 53.2) | 45.1 (44.7, 45.5) |
| Gansu | 45.6 (45.2, 46.0) | 41.3 (40.9, 41.6) |  | 45.4 (45.0, 45.8) | 41.3 (41.0, 41.7) |  | 47.6 (47.1, 48.1) | 42.6 (42.3, 43.0) |  | 50.0 (49.6, 50.5) | 44.5 (44.1, 44.9) |  | 52.2 (51.6, 52.7) | 46.5 (46.1, 46.9) |
| Guizhou | 41.2 (40.8, 41.6) | 38.8 (38.5, 39.2) |  | 41.6 (41.1, 42.0) | 38.8 (38.5, 39.2) |  | 43.7 (43.2, 44.1) | 40.0 (39.7, 40.4) |  | 45.2 (44.8, 45.7) | 41.2 (40.8, 41.5) |  | 47.6 (47.2, 48.1) | 43.5 (43.1, 43.9) |
| Hainan | 42.3 (41.9, 42.8) | 39.0 (38.6, 39.3) |  | 43.0 (42.6, 43.4) | 39.8 (39.5, 40.1) |  | 45.1 (44.6, 45.5) | 40.1 (39.7, 40.4) |  | 44.8 (44.4, 45.2) | 40.4 (40.1, 40.7) |  | 48.6 (48.1, 49.1) | 42.9 (42.5, 43.3) |
| Chongqing | 42.8 (42.4, 43.3) | 39.1 (38.7, 39.4) |  | 42.8 (42.5, 43.2) | 39.9 (39.6, 40.2) |  | 46.1 (45.6, 46.5) | 41.1 (40.8, 41.5) |  | 48.0 (47.6, 48.5) | 42.4 (42.1, 42.8) |  | 49.1 (48.6, 49.7) | 46.0 (45.6, 46.4) |
| Jiangsu | 48.2 (47.7, 48.7) | 43.0 (42.6, 43.3) |  | 51.5 (51.1, 51.9) | 45.2 (44.9, 45.5) |  | 51.5 (51.0, 51.9) | 44.7 (44.4, 45.1) |  | 52.7 (52.2, 53.2) | 46.0 (45.7, 46.4) |  | 54.4 (53.9, 55.0) | 47.0 (46.6, 47.4) |
| Jiangxi | 44.6 (44.2, 45.0) | 40.5 (40.1, 40.8) |  | 45.3 (44.8, 45.7) | 41.4 (41.1, 41.8) |  | 46.7 (46.2, 47.1) | 41.8 (41.4, 42.1) |  | 49.1 (48.7, 49.6) | 43.5 (43.1, 43.9) |  | 50.6 (50.1, 51.1) | 44.2 (43.8, 44.6) |
| Shanxi | 46.5 (46.0, 47.0) | 42.0 (41.6, 42.3) |  | 47.8 (47.3, 48.3) | 42.4 (42.0, 42.7) |  | 47.6 (47.0, 48.3) | 42.7 (42.2, 43.2) |  | 50.0 (49.5, 50.4) | 44.3 (43.9, 44.6) |  | 52.5 (51.9, 53.0) | 46.0 (45.6, 46.4) |
| Zhejiang | 47.2 (46.8, 47.6) | 42.8 (42.4, 43.1) |  | 48.1 (47.6, 48.5) | 42.5 (42.2, 42.8) |  | 49.9 (49.4, 50.4) | 43.6 (43.3, 44.0) |  | 51.5 (51.0, 52.0) | 44.5 (44.1, 44.8) |  | 53.1 (52.6, 53.6) | 45.6 (45.2, 46.0) |
| Tianjin | 51.3 (50.6, 51.9) | 45.2 (44.7, 45.7) |  | 52.9 (52.4, 53.4) | 48.9 (48.6, 49.2) |  | 52.0 (51.2, 52.7) | 45.7 (45.1, 46.2) |  | 56.9 (56.3, 57.5) | 48.0 (47.6, 48.5) |  | 57.0 (56.4, 57.6) | 49.2 (48.7, 49.7) |
| Beijing | 49.5 (49.0, 50.1) | 43.4 (43.0, 43.8) |  | 52.5 (51.9, 53.0) | 45.4 (45.0, 45.8) |  | 52.1 (51.4, 52.9) | 45.6 (45.0, 46.1) |  | 54.9 (54.3, 55.4) | 47.1 (46.6, 47.5) |  | 55.1 (54.6, 55.7) | 47.2 (46.8, 47.7) |
| Ningxia | 44.3 (43.9, 44.8) | 41.4 (41.0, 41.8) |  | 47.2 (46.7, 47.6) | 42.6 (42.2, 43.0) |  | 48.4 (47.9, 48.8) | 43.0 (42.6, 43.4) |  | 50.0 (49.5, 50.5) | 44.3 (43.9, 44.7) |  | 49.7 (49.1, 50.3) | 44.1 (43.6, 44.6) |
| Guangdong | 42.4 (42.0, 42.8) | 39.1 (38.8, 39.5) |  | 43.6 (43.2, 44.0) | 39.4 (39.1, 39.7) |  | 46.0 (45.6, 46.4) | 41.0 (40.7, 41.3) |  | 47.1 (46.7, 47.6) | 41.9 (41.6, 42.3) |  | 47.6 (47.2, 48.1) | 42.0 (41.7, 42.4) |
| Henan | 48.3 (47.8, 48.7) | 43.1 (42.8, 43.5) |  | 48.3 (47.9, 48.7) | 43.3 (43.0, 43.6) |  | 50.2 (49.7, 50.7) | 44.2 (43.9, 44.6) |  | 51.0 (50.5, 51.5) | 45.4 (45.0, 45.8) |  | 53.5 (52.9, 54.0) | 46.5 (46.1, 47.0) |
| Guangxi | 43.5 (43.1, 44.0) | 39.5 (39.1, 39.8) |  | 44.2 (43.7, 44.6) | 39.8 (39.5, 40.2) |  | 45.8 (45.3, 46.2) | 40.8 (40.5, 41.1) |  | 47.7 (47.2, 48.2) | 42.0 (41.7, 42.4) |  | 48.3 (47.8, 48.8) | 42.8 (42.4, 43.2) |
| Qinghai | 42.2 (41.5, 43.0) | 39.1 (38.4, 39.7) |  | 42.8 (42.4, 43.3) | 39.5 (39.1, 39.9) |  | 44.7 (44.2, 45.1) | 40.3 (39.9, 40.7) |  | 46.5 (46.0, 46.9) | 42.0 (41.6, 42.4) |  | 46.5 (45.9, 47.1) | 43.3 (42.8, 43.7) |
| Yunnan | 45.2 (44.7, 45.7) | 41.2 (40.8, 41.6) |  | 44.9 (44.4, 45.3) | 40.7 (40.4, 41.1) |  | 45.7 (45.2, 46.2) | 41.4 (41.0, 41.7) |  | 48.1 (47.6, 48.6) | 42.7 (42.4, 43.1) |  | 48.9 (48.4, 49.4) | 43.4 (43.1, 43.8) |
| Sichuan | 47.4 (47.1, 47.8) | 42.2 (41.9, 42.5) |  | 46.4 (46.0, 46.7) | 42.2 (42.0, 42.5) |  | 44.9 (44.4, 45.3) | 40.3 (40.0, 40.6) |  | 46.5 (46.1, 46.9) | 42.0 (41.7, 42.4) |  | 47.7 (47.2, 48.2) | 42.9 (42.5, 43.2) |

The 30 provinces of mainland China were ordered by absolute changes in weight for boys from 2000 to 2019 in descending order.

# Table S2: Handgrip strength (kg)/height^2^ (m^2^) by age, sex, and region of the Chinese National Survey on Students’ Constitution and Health, in 2000–2019

| Age | Region | 2000 | |  | 2005 | |  | 2010 | |  | 2014 | |  | 2019 | |
| --- | --- | --- | --- | --- | --- | --- | --- | --- | --- | --- | --- | --- | --- | --- | --- |
|  |  | Boys | Girls |  | Boys | Girls |  | Boys | Girls |  | Boys | Girls |  | Boys | Girls |
| 7 years | East | 4.4 (2.2) | 3.7 (2.1) |  | 6.4 (1.6) | 5.7 (1.6) |  | 5.8 (1.8) | 5.2 (1.6) |  | 6.4 (1.5) | 5.8 (1.4) |  | 6.5 (1.8) | 5.8 (1.5) |
|  | Centre | 4.9 (2.2) | 4.5 (2.3) |  | 6.3 (1.5) | 5.6 (1.4) |  | 6.9 (1.5) | 6.1 (1.4) |  | 6.5 (1.7) | 5.8 (1.6) |  | 6.7 (2.5) | 6.2 (2.9) |
|  | South | 5.2 (2.7) | 4.3 (2.3) |  | 6.0 (1.8) | 5.3 (1.7) |  | 6.3 (1.6) | 5.9 (3.0) |  | 6.4 (1.5) | 5.7 (1.4) |  | 6.5 (1.8) | 6.0 (1.5) |
|  | Southwest | 5.7 (2.0) | 4.8 (1.8) |  | 6.8 (2.1) | 6.0 (1.5) |  | 6.5 (1.5) | 5.8 (1.5) |  | 6.4 (1.5) | 5.6 (1.4) |  | 6.6 (1.7) | 5.9 (1.9) |
|  | North | 6.5 (3.4) | 5.6 (2.8) |  | 6.6 (1.8) | 5.8 (1.5) |  | 7.0 (1.5) | 6.2 (1.5) |  | 6.7 (1.6) | 5.9 (1.5) |  | 6.4 (1.5) | 5.8 (1.7) |
|  | Northeast | 5.7 (2.2) | 5.0 (2.3) |  | 7.2 (2.7) | 6.4 (2.8) |  | 6.6 (2.0) | 5.9 (2.5) |  | 6.6 (1.6) | 5.8 (1.5) |  | 6.5 (2.6) | 5.9 (1.8) |
|  | Northwest | 4.1 (2.3) | 3.3 (2.0) |  | 6.9 (1.8) | 5.9 (2.1) |  | 6.8 (1.9) | 6.0 (1.3) |  | 6.5 (1.5) | 5.8 (1.4) |  | 6.6 (1.9) | 5.7 (1.7) |
|  |  |  |  |  |  |  |  |  |  |  |  |  |  |  |  |
| 12 years | East | 7.8 (2.4) | 6.9 (2.6) |  | 9.5 (1.8) | 8.4 (1.6) |  | 9.4 (2.1) | 8.2 (1.8) |  | 9.6 (2.0) | 8.4 (1.8) |  | 9.3 (2.1) | 8.3 (1.8) |
|  | Centre | 8.3 (1.9) | 7.2 (1.8) |  | 9.6 (1.9) | 8.4 (1.6) |  | 10.1 (2.0) | 8.8 (1.7) |  | 10.1 (2.2) | 8.9 (1.8) |  | 9.3 (2.3) | 8.2 (2.2) |
|  | South | 8.6 (2.4) | 8.0 (2.4) |  | 8.5 (2.5) | 7.8 (2.3) |  | 9.4 (2.0) | 8.5 (1.7) |  | 9.4 (2.0) | 8.5 (1.7) |  | 9.3 (2.2) | 8.5 (1.9) |
|  | Southwest | 8.4 (2.0) | 7.6 (1.7) |  | 9.5 (1.9) | 8.5 (1.8) |  | 9.2 (1.9) | 8.5 (1.7) |  | 9.2 (1.8) | 8.3 (1.7) |  | 9.2 (1.9) | 8.7 (2.1) |
|  | North | 8.9 (2.6) | 7.7 (2.2) |  | 10.0 (2.1) | 8.5 (1.6) |  | 10.0 (2.0) | 8.8 (1.7) |  | 9.5 (2.0) | 8.5 (1.7) |  | 9.1 (2.1) | 8.1 (1.9) |
|  | Northeast | 9.4 (2.7) | 7.6 (2.1) |  | 9.4 (2.1) | 8.2 (2.0) |  | 9.5 (2.5) | 7.9 (2.5) |  | 9.3 (2.0) | 8.3 (1.7) |  | 9.3 (2.2) | 8.2 (1.7) |
|  | Northwest | 7.3 (2.6) | 6.3 (2.2) |  | 9.4 (2.0) | 8.2 (1.8) |  | 9.4 (1.9) | 8.1 (1.8) |  | 9.5 (1.9) | 8.3 (1.8) |  | 9.3 (2.5) | 8.4 (2.5) |
|  |  |  |  |  |  |  |  |  |  |  |  |  |  |  |  |
| 18 years | East | 13.8 (2.4) | 9.2 (2.0) |  | 14.5 (2.3) | 10.2 (1.9) |  | 14.7 (2.3) | 10.2 (1.9) |  | 14.0 (2.5) | 10.0 (1.8) |  | 13.0 (2.7) | 9.5 (2.0) |
|  | Centre | 14.3 (2.3) | 10.1 (2.0) |  | 14.7 (2.3) | 10.8 (1.9) |  | 15.2 (2.4) | 11.1 (1.9) |  | 15.2 (2.7) | 10.8 (2.3) |  | 13.5 (3.1) | 9.3 (2.5) |
|  | South | 14.2 (2.4) | 10.2 (2.6) |  | 14.6 (3.5) | 10.8 (2.5) |  | 14.3 (2.4) | 10.2 (2.0) |  | 14.6 (2.4) | 10.5 (2.0) |  | 13.7 (2.7) | 10.3 (2.1) |
|  | Southwest | 13.8 (3.3) | 9.9 (2.8) |  | 14.5 (2.7) | 10.6 (2.0) |  | 15.0 (2.3) | 10.9 (2.0) |  | 14.6 (2.3) | 10.2 (2.0) |  | 13.8 (2.5) | 10.1 (2.0) |
|  | North | 14.3 (2.5) | 9.9 (2.2) |  | 14.5 (2.5) | 10.4 (1.9) |  | 14.9 (2.6) | 10.6 (2.0) |  | 14.9 (2.6) | 10.6 (2.0) |  | 13.8 (2.4) | 9.9 (2.0) |
|  | Northeast | 13.3 (2.8) | 8.9 (2.2) |  | 14.1 (2.5) | 10.0 (2.0) |  | 13.9 (2.3) | 10.3 (2.1) |  | 14.3 (3.5) | 9.3 (2.3) |  | 13.3 (2.8) | 9.7 (2.3) |
|  | Northwest | 13.3 (2.7) | 9.0 (2.6) |  | 15.1 (3.1) | 10.5 (2.3) |  | 14.5 (2.3) | 10.3 (1.8) |  | 14.6 (2.3) | 10.1 (1.8) |  | 13.8 (2.3) | 9.8 (2.0) |
|  |  |  |  |  |  |  |  |  |  |  |  |  |  |  |  |
| 22 years | East | 14.6 (2.8) | 10.1 (2.5) |  | 15.3 (2.3) | 10.5 (2.0) |  | 15.0 (2.3) | 10.5 (1.8) |  | 14.8 (2.5) | 10.2 (1.9) |  | 14.1 (2.8) | 10.2 (2.1) |
|  | Centre | 14.4 (3.1) | 10.0 (2.4) |  | 14.9 (2.5) | 11.0 (2.1) |  | 14.9 (2.5) | 10.5 (2.1) |  | 16.8 (4.3) | 10.2 (3.0) |  | 14.4 (2.5) | 10.0 (2.0) |
|  | South | 14.2 (2.2) | 9.8 (2.0) |  | 14.9 (2.0) | 10.7 (1.8) |  | 15.5 (2.3) | 10.7 (2.3) |  | 14.6 (2.7) | 10.1 (2.1) |  | 14.2 (2.4) | 10.1 (1.8) |
|  | Southwest | 14.8 (2.3) | 10.0 (2.1) |  | 14.7 (2.6) | 10.4 (1.9) |  | 14.1 (3.2) | 10.6 (2.1) |  | 14.2 (2.4) | 9.9 (1.9) |  | 13.7 (2.8) | 9.8 (2.5) |
|  | North | 14.8 (2.4) | 9.4 (2.4) |  | 15.3 (2.4) | 10.5 (1.9) |  | 14.9 (2.4) | 10.3 (1.9) |  | 15.5 (2.6) | 11.0 (2.1) |  | 14.6 (2.5) | 10.3 (1.8) |
|  | Northeast | 15.0 (2.8) | 10.0 (2.6) |  | 15.7 (5.5) | 10.7 (4.0) |  | 14.3 (3.0) | 10.3 (2.2) |  | 14.6 (3.0) | 10.9 (2.7) |  | 13.7 (2.8) | 9.9 (2.5) |
|  | Northwest | 15.1 (2.5) | 10.0 (2.0) |  | 14.4 (2.6) | 10.8 (1.9) |  | 15.0 (3.1) | 10.1 (2.5) |  | 14.9 (2.4) | 10.5 (2.1) |  | 15.0 (3.2) | 10.3 (2.2) |
|  |  |  |  |  |  |  |  |  |  |  |  |  |  |  |  |
| 7, 22 years | East | 10.0 (4.4) | 7.5 (3.2) |  | 11.3 (3.7) | 8.8 (2.3) |  | 11.1 (3.9) | 8.6 (2.5) |  | 11.0 (3.6) | 8.6 (2.2) |  | 10.7 (3.5) | 8.5 (2.3) |
|  | Centre | 10.4 (4.2) | 7.8 (2.9) |  | 11.2 (3.8) | 8.9 (2.5) |  | 11.7 (3.7) | 9.2 (2.4) |  | 11.9 (4.3) | 9.0 (2.6) |  | 10.8 (3.7) | 8.5 (2.6) |
|  | South | 10.4 (4.1) | 8.1 (3.0) |  | 10.8 (4.3) | 8.5 (2.9) |  | 11.2 (3.9) | 8.8 (2.6) |  | 11.1 (3.6) | 8.8 (2.3) |  | 10.7 (3.6) | 8.7 (2.4) |
|  | Southwest | 11.2 (4.2) | 8.4 (2.9) |  | 11.3 (3.7) | 8.9 (2.5) |  | 11.1 (3.7) | 8.9 (2.4) |  | 11.0 (3.6) | 8.6 (2.3) |  | 10.6 (3.5) | 8.6 (2.4) |
|  | North | 10.8 (4.0) | 8.2 (2.8) |  | 11.7 (3.8) | 8.9 (2.4) |  | 11.7 (3.7) | 9.1 (2.3) |  | 11.4 (3.8) | 8.9 (2.4) |  | 10.8 (3.6) | 8.5 (2.4) |
|  | Northeast | 10.3 (3.8) | 7.8 (2.7) |  | 11.5 (3.8) | 8.8 (2.6) |  | 11.0 (3.7) | 8.6 (2.6) |  | 11.1 (3.9) | 8.6 (2.5) |  | 10.6 (3.5) | 8.3 (2.4) |
|  | Northwest | 9.9 (4.5) | 7.2 (3.1) |  | 11.4 (3.9) | 8.9 (2.6) |  | 11.2 (3.7) | 8.7 (2.4) |  | 11.3 (3.7) | 8.7 (2.4) |  | 10.7 (3.6) | 8.5 (2.5) |

Data are means (standard deviations). The 30 provinces of mainland China were grouped into seven regions: East (Shanghai, Jiangsu, Zhejiang, Anhui, Fujian, Jiangxi, Shandong), Centre (Henan, Hubei, Hunan), South (Guangdong, Guangxi, Hainan), Southwest (Chongqing, Sichuan, Guizhou, Yunnan), North (Beijing, Tianjin, Hebei, Shanxi, Inner Mongolia), Northeast (Liaoning, Jilin, Heilongjiang), and Northwest (Shaanxi, Gansu, Qinghai, Ningxia, Xinjiang).

# Table S3: Differences in change of muscle strength and weight by age and sex of the Chinese National Survey on Students’ Constitution and Health, in 2000–2010, 2010–2019

|  | **Period** | **Age1, years** | **Age2, years** | **Differences and 95 CIs in absolute change** | ***P* values*** |
| --- | --- | --- | --- | --- | --- |
| **Boy** |  |  |  |  |  |
| **Handgrip strength** |  |  |  |  |  |
|  | 2000-2010 | 7 | 12 | -2.1 (-2.3, -1.9) | 0.000 |
|  | 2000-2010 | 7 | 18 | -1.5 (-1.7, -1.3) | 0.000 |
|  | 2000-2010 | 12 | 22 | 1.9 (1.6, 2.3) | 0.000 |
|  | 2000-2010 | 18 | 22 | 1.3 (1.0, 1.7) | 0.000 |
|  | 2000-2010 | 12 | 18 | 0.6 (0.4, 0.9) | 0.000 |
|  | 2000-2010 | 7 | 22 | -0.2 (-0.5, 0.1) | 1.000 |
|  | 2010-2019 | 7 | 18 | 3.1 (2.8, 3.3) | 0.000 |
|  | 2010-2019 | 7 | 22 | 1.6 (1.3, 2.0) | 0.000 |
|  | 2010-2019 | 12 | 18 | 3.3 (3.0, 3.6) | 0.000 |
|  | 2010-2019 | 12 | 22 | 1.8 (1.4, 2.2) | 0.000 |
|  | 2010-2019 | 18 | 22 | -1.5 (-1.8, -1.1) | 0.000 |
|  | 2010-2019 | 7 | 12 | -0.2 (-0.4, 0.0) | 0.452 |
| **Standing broad jump** |  |  |  |  |  |
|  | 2000-2010 | 7 | 22 | 5.6 (4.7, 6.5) | 0.000 |
|  | 2000-2010 | 18 | 22 | 3.4 (2.4, 4.3) | 0.000 |
|  | 2000-2010 | 7 | 12 | 2.7 (1.9, 3.6) | 0.000 |
|  | 2000-2010 | 12 | 22 | 2.9 (1.9, 3.9) | 0.000 |
|  | 2000-2010 | 7 | 18 | 2.2 (1.4, 3.1) | 0.000 |
|  | 2000-2010 | 12 | 18 | -0.5 (-1.4, 0.4) | 1.000 |
|  | 2010-2019 | 7 | 18 | 4.1 (3.3, 5.0) | 0.000 |
|  | 2010-2019 | 7 | 22 | 4.6 (3.6, 5.6) | 0.000 |
|  | 2010-2019 | 12 | 22 | 4.1 (3.0, 5.2) | 0.000 |
|  | 2010-2019 | 12 | 18 | 3.6 (2.6, 4.6) | 0.000 |
|  | 2010-2019 | 7 | 12 | 0.5 (-0.4, 1.4) | 1.000 |
|  | 2010-2019 | 18 | 22 | 0.5 (-0.6, 1.6) | 1.000 |
| **Weight** |  |  |  |  |  |
|  | 2000-2010 | 7 | 12 | -2.3 (-2.6, -1.9) | 0.000 |
|  | 2000-2010 | 12 | 18 | 2.8 (2.4, 3.2) | 0.000 |
|  | 2000-2010 | 18 | 22 | -1.9 (-2.4, -1.5) | 0.000 |
|  | 2000-2010 | 7 | 22 | -1.4 (-1.8, -1.1) | 0.000 |
|  | 2000-2010 | 12 | 22 | 0.9 (0.4, 1.3) | 0.001 |
|  | 2000-2010 | 7 | 18 | 0.5 (0.2, 0.8) | 0.013 |
|  | 2010-2019 | 7 | 12 | -3.6 (-4.0, -3.2) | 0.000 |
|  | 2010-2019 | 7 | 18 | -3.1 (-3.5, -2.8) | 0.000 |
|  | 2010-2019 | 7 | 22 | -2.4 (-2.9, -2.0) | 0.000 |
|  | 2010-2019 | 12 | 22 | 1.2 (0.6, 1.7) | 0.000 |
|  | 2010-2019 | 18 | 22 | 0.7 (0.2, 1.3) | 0.058 |
|  | 2010-2019 | 12 | 18 | 0.5 (0, 1.0) | 0.446 |
| **Girl** |  |  |  |  |  |
| **Handgrip strength** |  |  |  |  |  |
|  | 2000-2010 | 7 | 12 | -1.9 (-2.1, -1.8) | 0.000 |
|  | 2000-2010 | 7 | 18 | -1.5 (-1.7, -1.3) | 0.000 |
|  | 2000-2010 | 12 | 22 | 1.3 (1.0, 1.6) | 0.000 |
|  | 2000-2010 | 18 | 22 | 0.8 (0.6, 1.1) | 0.000 |
|  | 2000-2010 | 7 | 22 | -0.6 (-0.9, -0.4) | 0.000 |
|  | 2000-2010 | 12 | 18 | 0.5 (0.2, 0.7) | 0.000 |
|  | 2010-2019 | 7 | 18 | 1.8 (1.6, 2.0) | 0.000 |
|  | 2010-2019 | 12 | 18 | 2 (1.8, 2.2) | 0.000 |
|  | 2010-2019 | 12 | 22 | 1.1 (0.9, 1.4) | 0.000 |
|  | 2010-2019 | 7 | 22 | 1.0 (0.7, 1.2) | 0.000 |
|  | 2010-2019 | 18 | 22 | -0.8 (-1.1, -0.6) | 0.000 |
|  | 2010-2019 | 7 | 12 | -0.2 (-0.4, 0.0) | 0.194 |
| **Standing broad jump** |  |  |  |  |  |
|  | 2000-2010 | 7 | 12 | 4.7 (3.9, 5.4) | 0.000 |
|  | 2000-2010 | 7 | 18 | 5.1 (4.3, 5.8) | 0.000 |
|  | 2000-2010 | 7 | 22 | 6.1 (5.2, 6.9) | 0.000 |
|  | 2000-2010 | 12 | 22 | 1.4 (0.5, 2.3) | 0.009 |
|  | 2000-2010 | 18 | 22 | 1.0 (0.1, 1.9) | 0.147 |
|  | 2000-2010 | 12 | 18 | 0.4 (-0.4, 1.2) | 1.000 |
|  | 2010-2019 | 7 | 12 | 2.3 (1.5, 3.1) | 0.000 |
|  | 2010-2019 | 7 | 18 | 1.3 (0.5, 2.1) | 0.008 |
|  | 2010-2019 | 7 | 22 | 1.3 (0.4, 2.2) | 0.019 |
|  | 2010-2019 | 12 | 18 | -1.0 (-1.9, -0.2) | 0.118 |
|  | 2010-2019 | 12 | 22 | -1.0 (-1.9, -0.1) | 0.230 |
|  | 2010-2019 | 18 | 22 | 0 (-0.9, 1.0) | 1.000 |
| **Weight** |  |  |  |  |  |
|  | 2000-2010 | 7 | 18 | 1.4 (1.1, 1.6) | 0.000 |
|  | 2000-2010 | 7 | 22 | 1.2 (0.9, 1.5) | 0.000 |
|  | 2000-2010 | 12 | 18 | 2.5 (2.2, 2.9) | 0.000 |
|  | 2000-2010 | 12 | 22 | 2.4 (2.0, 2.7) | 0.000 |
|  | 2000-2010 | 7 | 12 | -1.2 (-1.5, -0.9) | 0.000 |
|  | 2000-2010 | 18 | 22 | -0.2 (-0.5, 0.1) | 1.000 |
|  | 2010-2019 | 7 | 12 | -2.8 (-3.2, -2.5) | 0.000 |
|  | 2010-2019 | 7 | 18 | -1.3 (-1.6, -1.0) | 0.000 |
|  | 2010-2019 | 12 | 18 | 1.5 (1.2, 1.9) | 0.000 |
|  | 2010-2019 | 12 | 22 | 1.6 (1.2, 2.0) | 0.000 |
|  | 2010-2019 | 7 | 22 | -1.2 (-1.6, -0.9) | 0.000 |
|  | 2010-2019 | 18 | 22 | 0.1 (-0.3, 0.5) | 1.000 |

CI = confidence interval. ^*^Pairwise age group comparisons with Bonferroni-adjusted *P* values.

# Figure S1: Temporal trends in handgrip strength (A, B), standing broad jump (C, D), and weight (E, F) by age and sex in mainland China, in 2000–2019


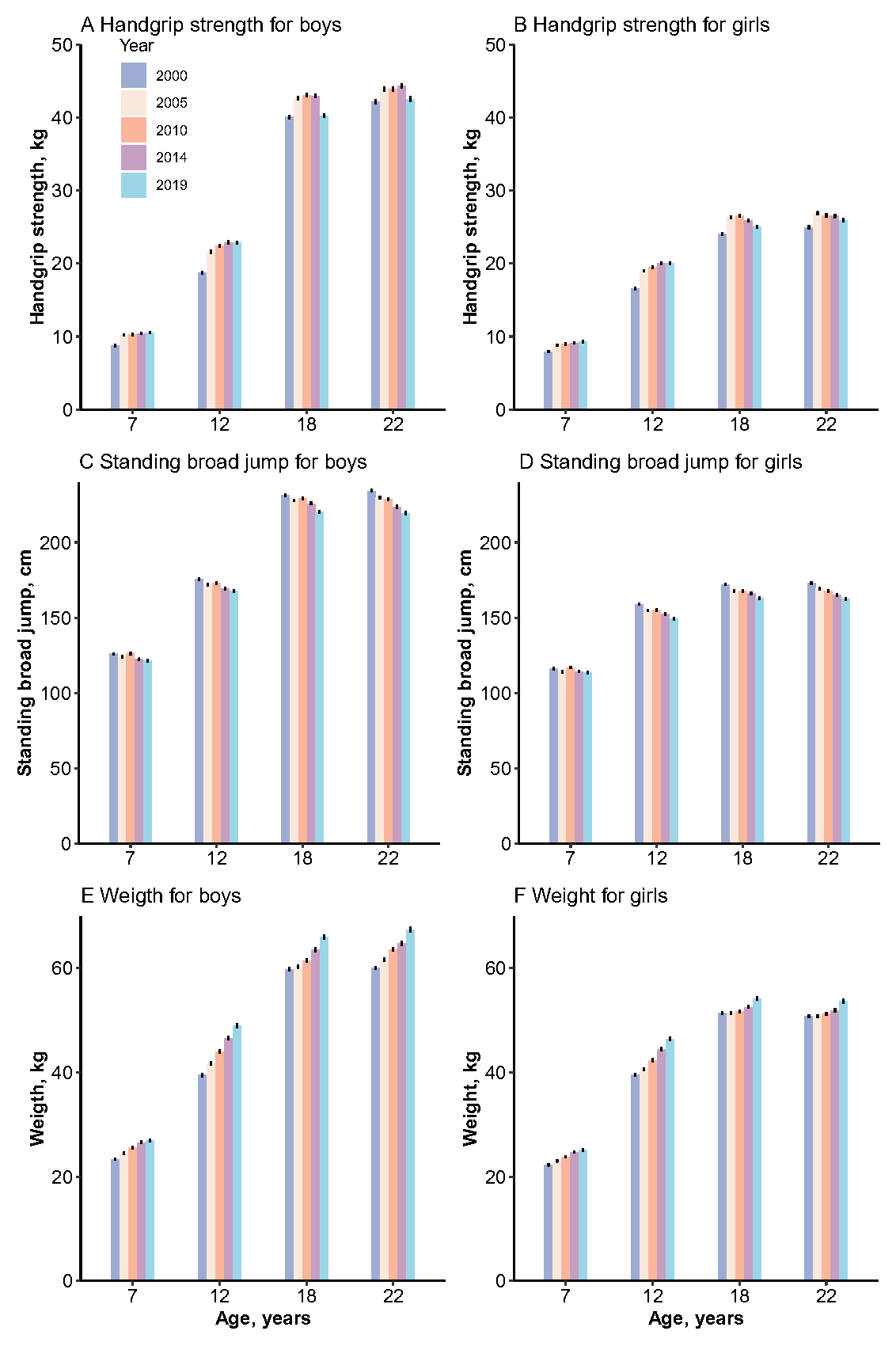


Note: The black lines indicate 95% confidence intervals.

# Figure S2: Absolute changes in handgrip strength (A, B), standing broad jump (C, D), and weight (E, F) by age and sex in mainland China, in 2000–2019


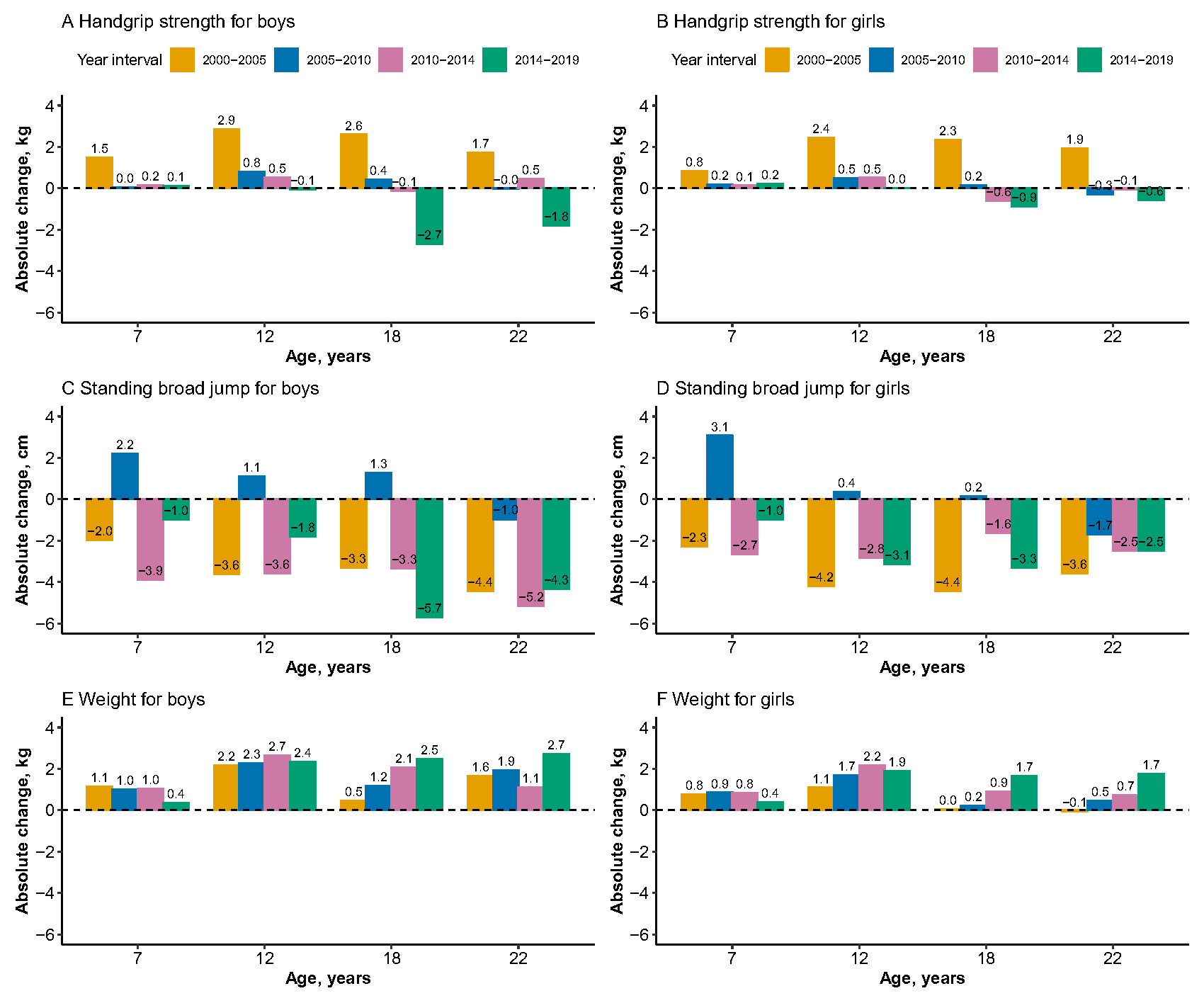


# Figure S3: Provincial changes in handgrip strength (A, B), standing broad jump (C, D), and weight (E, F) among students aged 7–22 years by sex, in 2010–2019


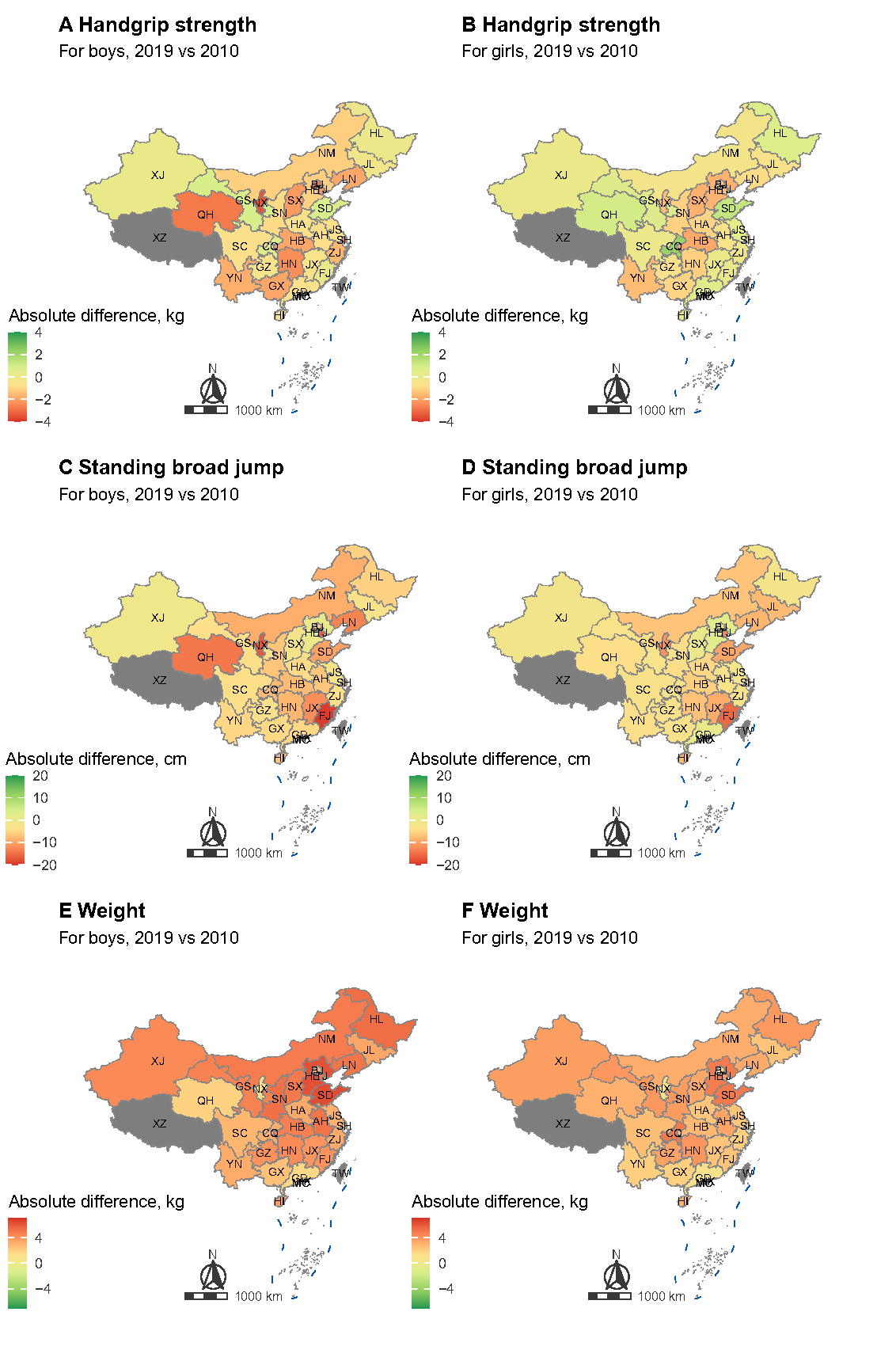


AH=Anhui; BJ=Beijing; CQ=Chongqing; FJ=Fujian; GD=Guangdong; GS=Gansu; GX=Guangxi; GZ=Guizhou; HA=Henan; HB=Hubei; HE=Hebei; HI=Hainan; HK=Hong Kong; HL=Heilongjiang; HN=Hunan; JL=Jilin; JS=Jiangsu; JX=Jiangxi; LN=Liaoning; MO=Macao; NM=Inner Mongolia; NX=Ningxia; QH=Qinghai; SC=Sichuan; SD=Shandong; SH=Shanghai; SN=Shaanxi; SX=Shanxi; TJ=Tianjin; TW=Taiwan; XJ=Xinjiang; XZ=Tibet; YN=Yunnan; ZJ=Zhejiang· Data for Tibet, Hong Kong, Macao, and Taiwan are not available.

Figure S4. Association between muscle strength and weight among students aged 7–22 years by sex (A and C for boys; B and D for girls) in mainland China, in 2000–2019.
